# Biofertilizer use in the United States: definition, regulation, and prospects

**DOI:** 10.1007/s00253-024-13347-4

**Published:** 2024-11-12

**Authors:** Flavia Santos, Suraj Melkani, Christiane Oliveira-Paiva, Daniel Bini, Kiran Pavuluri, Luke Gatiboni, Anik Mahmud, Maria Torres, Eric McLamore, Jehangir H. Bhadha

**Affiliations:** 1 Soil, Water & Ecosystem Sciences Department, University of Florida, Belle Glade, FL USA; 2 Embrapa Maize and Sorghum, Sete Lagoas, Minas Gerais Brazil; 3 International Fertilizer Development Center, Muscle Shoals, AL USA; 4 North Carolina State Extension, North Carolina State University, Raleigh, NC USA; 5 Plant and Environmental Sciences, Clemson University, Clemson, SC USA; 6 Agricultural Sciences, Clemson University, Clemson, SC USA; 7 Environmental Engineering and Earth Sciences, Clemson University, Clemson, SC USA

**Keywords:** Biological, Biostimulant, Microorganisms, Inoculant, Soil microbiology

## Abstract

**Abstract:**

The increasing demand for sustainable food production has driven a surge in the use and commercialization of biological inputs, including biofertilizers. In this context, biofertilizers offer potential benefits for nutrient use efficiency, crop yield and sustainability. However, inconsistent definition of the term “biofertilizer” and regulations, particularly in the USA, hinder market growth and consumer confidence. While the European Union, and countries like Brazil, India, and China have made progress in this area, the USA market, projected to exceed $1 billion by 2029, lacks clear guidelines for biofertilizer production and sale. The USA market is dominated by *Rhizobium* genus, *Mycorrhizae* fungi, and *Azospirillum* species and based products targeting various crops. Although there is a growing and promising market for the use of biofertilizers, there are still many challenges to overcome, and to fully realize the potential of biofertilizers, future research should focus on modes of action, specific claims, and robust regulations that must be established.

**Key points:**

• *The term “biofertilizer” lacks a universally accepted definition*

• *It is necessary establishing a national regulation for biofertilizers in the USA*

• *The biofertilizer market is growing fast and the biggest one is in America*

**Supplementary Information:**

The online version contains supplementary material available at 10.1007/s00253-024-13347-4.

## Introduction

The world faces multiple challenges, including climate change, food security, and geopolitical trade crises, alongside growing demand for sustainable food production practices that harmonize with environmental goals. Global agreements such as COP21 (i.e., Paris Agreement) and COP26 (i.e., Glasgow Climate Pact) emphasize the need for sustainable food production, based on ESG (Environmental, Social, and Governance) principles and circular economy models, primarily aiming to reduce greenhouse gas emissions.

Traditional agricultural models rely heavily on synthetic fertilizers and pesticides (Sing and Kumar 2024), which, despite their benefits in ensuring crop yields, can lead to environmental issues such as soil and water contamination and adverse health effects due to eutrophication and harmful algal blooms (Rehman et al. 2021).

A sustainable and more environmentally friendly alternative to traditional practices is the use of biofertilizers in agriculture. Biofertilizers can boost plant growth and yield by 10–40% (Stewart and Roberts 2012), and they can be a low-cost source to improve nutrient use efficiency. Aloo et al. (2022) reported that the application of biofertilizers can improve the yield of various crops by 25% and reduce the nitrogen (N) requirement by 50% and phosphorus (P) requirement by 25% in agriculture. Therefore, biofertilizer can improve soil and plant health. Better yield (above and belowground plant biomass) and a greater mass of beneficial microorganisms permit more organic matter to be incorporated into the soil that can help to improve soil structure, increase the availability and retention of nutrients and retain moisture (Kumar et al. 2022). Well-nourished plants, with more root production, are healthier and more resistant to attacks by pests, diseases and drought damage.

The term biofertilizer has been refined because of a better understanding of the soil–plant-microorganism interaction. In a review carried out by Malusá et al. (2017), initially a biofertilizer only comprised of rhizospheric organisms capable of improving the use of soil nutrients, but not replacing them (Okon and Labandera-Gonzalez 1994). More recent concepts indicate that biofertilizers, also known as inoculants, bioinoculants or bioformulations, are products composed of beneficial microorganisms, in either their active form or inactive forms, formulated with a single strain or in a mixed form, capable of colonizing the rhizosphere or the internal tissues of plants (Fasusi et al. 2021; Ibáñez et al. 2023). In turn, these microorganisms can act by promoting improvements in the availability of nutrients (N, P, and potassium (K), among others) from the soil and acquisition by plants, resulting in greater productivity of important crops (Malusá et al. 2017; Chaudhary et al. 2022).

As well as the biofertilizer definition, progress needs to be made regarding the legislation involving these products, as there are still gaps and inconsistencies depending on the country of its origin and application. According to the Biological Products Industry Alliance—BPIA (2023) some countries such as China, India, and Brazil have established laws with well-defined criteria on the use of biofertilizers and microbial inoculants, regarding production, labelling, quality control and other characteristics, while others, such as the European Union and the USA, are moving in this direction. India is probably the country with the most complete legal framework for biofertilizers and a good legal approach is important for producers who want to market them (Malusá and Vassilev 2014; BPIA 2023).

Due to the potential and market demand for the use of biofertilizers in agriculture, the biofertilizer market has been growing in impressive numbers. In the USA, the biofertilizers’ market size value in 2024, considering nitrogen-fixing, phosphate and K solubilizing and mobilizing biofertilizers, is estimated at approximately $3.55 billion and projected to grow to $4.47 billion by 2026, which means a high market value and an increase of 26% over two years (Source: Related Journals, Annual Reports, Press Releases, Company Publications, Company Websites, Expert Interviews, and MarketsandMarkets Analysis). However, while the importance of this input is growing, there is still ambiguity related to the definition of what are biofertilizers, their efficacy, and issues related to regulation and registration, which have resulted in a sluggish adoption of products within the USA.

In that sense, there is a need for a clear definition of the term “biofertilizer” and clarity in legislation. As highlighted in the Scope Newsletter (2024) the dialogue with industry, research and standard experts and other stakeholders confirmed the need to ensure market and regulatory clarity and user information and also a methodology to validate such claims.

This review is aimed at exploring the current state of biofertilizer use in the USA, focusing on its definition, regulation, marketability, adoption, and prospects.

## Material and methods

An extensive literature review was carried out on topics related to the definition of the term biofertilizer, regulation, adoption, and perspectives in the USA, compared to other countries. A database has been added to the existing textual database in this paper to provide a greater knowledge about the biofertilizer market in the USA and further information. The database came from independent and free data Telus Agronomy site (2024), which listed 235 biological products classified into two groups: inoculants and microbial stimulants. These products were organized by function/mechanism involved, brand name, registrant, ingredients, crops for which the product is indicated, physical state/form, and states where the product is approved.

### Biofertilizer definition and regulation in the USA

The words biofertilizers, biocontrol, and biostimulants are all used interchangeably in some cases, which cause confusion, because there is no established consensus on a clear and objective definition of the term biofertilizer, which impacts the market. There is some overlap in market information between biofertilizers and biostimulants, with biostimulants occasionally encompassing microbial inoculants that are also classified as biofertilizers. Plant biostimulants are materials that contain substance(s) and/or microorganisms whose function, when applied to plants or the rhizosphere, is to stimulate natural processes to enhance/benefit nutrient uptake, nutrient efficiency, tolerance to abiotic stress, and/or crop quality, independent of its nutrient. These beneficial properties are also often claimed for biofertilizer products, so for clarity, this review is focused only on microbial-based inoculants (O’Callaghan et al. 2022) (Table S1, Figures S1 A and B, S2, S3).

The term biofertilizer has evolved, focusing on beneficial microorganisms for plants and trying to separate it from other products used in agriculture, such as biopesticides (biological products to combat pests) and biostimulants (products that include substances produced by microorganisms or other beings) (Mitter et al. 2021a, b) (Table S2 and Figures S4 and S5). In this case, microorganisms that promote plant growth through biofungicidal effects, bionematocides, bioinsecticides, or any other products with a similar activity that favor plant health are generally defined as biopesticides, not biofertilizers (Vessey 2003).

Malusá and Vassilev (2014) highlight that the inconsistency in the definition of the term “biofertilizer,” in some cases, is due to the different kinds of microorganisms utilized to improve plant nutrition (*fungi* or *bacteria*) and the different mechanisms involved in the interaction between plant-soil-microorganisms. This has created some confusion in the market for microbial-based products for plant nutrition. These authors deal with the technical definition and the legal definition of the term biofertilizer, highlighting that technical definition of the term “biofertilizer” has been changed in different ways during the past 20 years.

Several authors (Kloepper 1993; Vessey 2003; Somers et al. 2004) state that when it comes to mechanisms of action, definitions can vary between bioenrichers or phytostimulators for microorganisms that promote plant growth and rhizomediators for those that degrade organic pollutants; also the same microorganism can express multiple mechanisms of action, making the definition merely theoretical. Vessey (2003) defined biofertilizer as “a substance which contains living microorganisms which when applied to seed, plant surfaces, or soil, colonizes the rhizosphere or the interior of the plant and promotes growth by increasing the supply or availability of primary nutrients to the host plant.” Malusá and Vassilev (2014) argue that the term “biofertilizer” should refer to a product that is ready to be marketed; i.e., it is the formulated product containing the microorganisms that are applied to the plant or soil (the authors also point out that a biofertilizer should not be confused with organic and/or mineral fertilizer or as a synonym for the formulations that define different types of organic fertilizers (e.g., compost and plant extracts) or biostimulants derived from microorganisms (e.g., products containing dead microbial cells, extracts from microbial cultures, etc.)) (Table S3).

du Jardin (2015) defines biofertilizers as a subcategory of biostimulants, but the definition of the two terms is still confusing: biostimulants are “any substances or microorganisms applied to plants with the aim to enhance nutrition efficiency, abiotic stress tolerance and/or crop quality traits, regardless of its nutrients content” and biofertilizers are “any bacterial or fungal inoculants applied to plants with the aim to increase the availability of nutrients and their utilization by plants, regardless of the nutrient content of the inoculant itself.” Dineshkumar et al. (2018) later proposed a modified definition of biofertilizers as “products (carrier or liquid based) containing living or dormant microbes (*bacteria*, *actinomycetes*, *fungi*, *algae*) alone or in combination, which help in *fixing atmospheric N* or *solubilizers soil nutrients* in addition to the secretion of growth promoting substances for enhancing crop growth and yield.” Kumar et al. (2022) provided a simple definition: biofertilizers are the formulation of living or latent cells of microbes, which provides an additional advantage in nutrient uptake and plant performance in the rhizosphere and the authors divide them into six different groups: (i) *N fixing microbes*, (ii/iii) *phosphorus (P)/K solubilizing and mobilizing microbes*, (iv) *zinc solubilizing microbes*, (v) *sulfur-oxidizing microbes*, and (vi) *plant promoting rhizobacteria*. This definition is similar to Shahwar et al. (2023) which biofertilizer refers to a wide range of products, containing living or dormant microorganisms and helps to improve the growth and yield of administered crops.

More recently, Daniel et al. (2022) defined biofertilizers as organic products that contain specific microorganisms obtained from plant roots and root zones, and they colonize the environment of the rhizosphere and the interior of the plant to promote plant growth. The authors divide biofertilizers into groups based on their functions (*nitrogen-fixing*,* P-solubilizing and mobilizing*,* K-solubilizing and K-mobilizing*, *micronutrient*, and *plant growth-promoting*), and mechanisms of action (increase the amount of N in the soil by fixing atmospheric N and making it available to plants, dissolve bound phosphates, secrete organic acids and lower soil pH by converting insoluble forms of P in the soil into soluble forms, produce hormones that encourage root growth, etc.). The most used biofertilizers are *N-fixers, K-solubilizers*, *P-solubilizers*, and *plant growth-promoting rhizobacteria* (PGPR) (Nosheen et al. 2021).

In addition to the differences in the definition of the term biofertilizer, there are different divisions within the group of biological products. Mercier (2023), Senior Policy Adviser at the Farm Journal Foundation, in an Op-ed titled “The use of Biologicals in agriculture,” divides biological inputs into two categories: biostimulants and biopesticides, with biofertilizers falling into the group of biostimulants, or enhancers, that are substances that enhance plant growth, health, and productivity or provide other direct or indirect benefits to a plant’s development.

The Plant Biostimulant Act of 2023 (BPIA 2023) also divides biological into two groups: biopesticides and biostimulants, and includes the biofertilizers within the latter group, that is defined as “Plant biostimulant meaning a substance, microorganism, or mixture thereof, that, when applied to seeds, plants, the rhizosphere, soil, or other growth media, act to support a plant’s natural processes independently of the biostimulants nutrient content, thereby improving nutrient availability, uptake or use efficiency, tolerance to abiotic stress, and consequent growth, development, quality, or yield.” The EPA Act 2023 states that a plant biostimulant shall not be subject to regulation under this Act.

More recently, Singh and Kumar (2024) divided biological products into three groups: bioinoculants—preparations containing single or consortia of microorganisms when introduced to the crop, and are dedicated to carrying out particular functions like, growth promotion, or biocontrol (Maitra et al. 2021); biofertilizer—a wide range of products, containing living or dormant microorganisms and helps to improve the growth and yield of administered crops (Shahwar et al. 2023), and biopesticides—products enriched with microbial inoculants capable of overcoming the stress caused due to phytopathogens and can promote the health of the crop (Yadav and Sarkar 2019).

Dunham (2015) also divides biological products into three groups: (i) biopesticides—derived from natural materials, such as plants, bacteria and certain minerals; (ii) biostimulants—seaweed extracts, organic acids, microbials, primarily bacteria, often used as seed or soil treatment to aid in nutrient assimilation; and (iii) biofertilizers—microbials used to enhance nutrient uptake from soil (*N-fixing bacteria, solubilizing of P and K*, and *mobilizers of zinc, sulfur*, and *mycorrhizal fungi*).

The Mordor Intelligence (2024) divides biological products in: organic fertilizers, biofertilizers, biostimulants, biopesticides, and biocontrol agents. It defines biofertilizers as organic substances with living microorganisms that promote plant growth. It is important to note that biofertilizer and organic fertilizer are different. Organic fertilizers are natural compounds of various kinds that contain carbon molecules, such as animal or plant remains. Therefore, they are derived from biological or living materials and take longer time to release the nutrient in the soil and come in the different forms such as manure derived from livestock such as cows, chickens, goats, and others; green manure which are obtained from young plants, especially different type of legumes; and compost derived from agricultural that is waste organic material such as straw, corn stalks, or decomposed waste (Sharma and Chetani 2017).

In this regard, du Jardin (2015) and Backer et al. (2018) highlighted that the absence of a standardized legal and regulatory definition for “plant biostimulants” is the primary reason behind the lack of a globally coordinated uniform regulatory policy, and the regulation of non-control products, as biostimulants or biofertilizers, in the USA is the responsibility of each state, in a way similar to fertilizers, and federal regulation is under discussion at the EPA (Environmental Protection Agency) (Ravensberg 2017; Caldwell and Fife 2023). On the contrary, pesticides are subject to regulation under the Federal Insecticide, Fungicide, and Rodenticide Act (FIFRA) as well as the plant regulator products (the substance or mixture of substances, through physiological action accelerates or retards the rate of plant growth; accelerates or retards the rate of plant maturation; or otherwise alters the behavior of plants or the produce thereof (Environmental Protection Agency-EPA 2020).

BPIA (2023) has highlighted the importance to develop a uniform, national definition for biostimulants (and biofertilizer is included in this class), considering its use across multiple agencies and sectors, such as federal and state agencies, academia, suppliers, regulators, and most importantly the end users or the farmers, because if a product is not defined somewhere in law or regulation, they do not exist.

As biofertilizers are largely unregulated, many small companies currently persist in the market; and the market will likely become more consolidated when regulations are imposed (Dunham 2015) and currently, in the USA, the industry of biofertilizers only has two paths to market, either via state fertilizer regulations, making claims only allowed within the context of fertilizer regulations, or via biostimulant claims (BPIA 2023). Conversely, EPA is very reluctant to allow products to make non-pesticidal “plant health” claims on their label under FIFRA (Federal Insecticide, Fungicide, and Rodenticide Act). In many instances, biostimulant companies are compelled to add non-essential (to their intended mode of action) nutrients to their products to fit their products into a particular state’s fertilizer approval process.

In response to these issues, BPIA (2023) has outlined key objectives for the industry, which include: (1) establishing a national legal definition for plant biostimulants through legislation or regulation; (2) permitting the use of the term “biostimulant” on product labels and marketing materials; (3) allowing the inclusion of validated biostimulant claims on product labels; (4) creating a clear, consistent, and predictable marketing process; (5) implementing a standardized label format across all 50 states; (6) ensuring science-based safety assessments for biostimulant products; (7) enabling active ingredients (A.I.) to be registered for dual uses in the market, similar to the European Union, where A.I.s can be classified as FIFRA pesticides at higher application rates or different use patterns when supported by data; and (8) achieving global consistency and harmonization for biostimulants. The document highlighted that these steps would establish credibility for the industry and for biostimulant products and would allow the industry, agriculture, in general, and USA rural economies to enjoy the benefits the rest of the world is experiencing with the introduction of biostimulants into farming practices (BPIA 2023). As mentioned in the document biostimulants are now, globally, the single largest input sector for agriculture and isn’t it time for the USA to establish a path to market for biostimulants and enjoy these benefits? (BPIA 2023).

### Biofertilizer definition and regulation outside the USA

The microorganisms that make up biofertilizers can have different origins and functions and can be classified according to the following criteria: microbial group, colonization environment, and microbial function for plant growth (Ibáñez et al. 2023). The main microbial groups for biofertilizers are *bacteria* and *fungi*. For example, species of the genus *Rhizobium*, *Bacillus*, *Pseudomonas*, and *Azotobacter* are the main bacteria that formulate biofertilizers in many countries, acting mainly in functions related to biological nitrogen fixation, phosphate solubilization and plant growth promotion (Aloo et al. 2022; Patel et al. 2023). Therefore, classification based on the functions of microorganisms, such as biological N fixation, P and K solubilization, phytohormone production, and siderophore production, among others, helps to identify specific groups, simplifying their use. However, many microorganisms are multifunctional; i.e., they can present various mechanisms that are beneficial to plants (Chaudhary et al. 2022; Ibáñez et al. 2023), a fact that can add value to biofertilizers that present additional mechanisms. An example of multifunctionality is *arbuscular mycorrhizal fungi* (AMF) involved in phosphate mobilization and plant protection (Patel et al. 2023). There are also *plant growth-promoting rhizobacteria* (PGPR), such as *Azospirillum brasiliensis*, which can be efficient at fixing N and producing phytohormones. Last but not least, the classification concerning the colonization environment separates biofertilizers formulated with rhizospheric (around the root), endophytic (inside the root), phyllospheric (aerial part of the plants), or free-living (non-rhizospheric regions of the soil) microorganisms (Ibáñez et al. 2023).

Based on technical criteria, some countries such as China, India, and Brazil have established laws on the use of biofertilizers and microbial inoculants, relating to production, labelling, quality control and other characteristics. India has long included the term “biofertilizer” in the law and created an appropriate regulation in Sect. 3 of the Essential Commodities Act, 1955 and Fertilizers (Control) Order, 1985. In this case, the Indian Ministry of Agriculture added biofertilizers to the Essential List. In Indian law, a biofertilizer is defined as “the product containing carrier-based living microorganisms (solid or liquid) which are useful for agriculture in terms of N fixation, P solubilization or nutrient mobilization, to increase the productivity of the soil and/or crop.” In Brazil, the term biofertilizer was first highlighted in law in 1980 and updated in 2004 and 2014 (DECREE No. 8.384, OF DECEMBER 29, 2014), with definitions on the inspection and supervision of fertilizer production and trade.

In Brazilian legislation, the term “biofertilizer” is more general, defined as “a product containing an active principle or organic agent, free of pesticides, capable of acting directly or indirectly on all or part of cultivated plants, increasing their productivity, without taking into account its hormonal or stimulant value.” The term “inoculant” refers to microorganisms, defined as “a product containing microorganisms that act favorably on plant growth.”

In Europe, the previous EU Regulation 2003/2003 established guidelines solely for inorganic fertilizers and liming agents. In contrast, the new EU Regulation 2019/1009 introduces up to seven Product Function Categories (PFCs) defined by the specific functions of each product. These categories include PFC 1 for fertilizers (covering inorganic, organic, and organo-mineral types); PFC 2 for liming materials; PFC 3 for soil improvers; PFC 4 for growing mediums; PFC 5 for inhibitors; PFC 6 for plant biostimulants; and PFC 7 for fertilizing product blends. Notably, the new framework differentiates between microbial biostimulants (PFC 6, A) and non-microbial biostimulants (PFC 6, B) (Regulation (EU) 2019/1009).

According to the rule, biostimulants for plants are defined as “a product that stimulates the processes of plant nutrition, regardless of the nutrient content of the product, with the sole aim of improving at least one of the following characteristics of plants or their rhizosphere: a) efficiency in the use of nutrients; b) tolerance to water stress; c) quality characteristics; d) availability of nutrients in the soil or rhizosphere” (Regulation (EU) 2019/1009).

The term biostimulant used by the European community follows the definition formulated by du Jardin (2015), who redefined the term as “any substance (e.g. amino acids, humic substances, phytohormones, among others) or microorganism applied to plants with the aim of increasing nutritional efficiency, abiotic stress tolerance and/or crop quality characteristics, regardless of their nutrient content” (du Jardin 2015). According to the European document, biostimulants must follow microbiological and chemical quality standards, and product labeling with information on the type of microorganisms, doses, intended function, rules of use, and possible sensitivity reactions. In addition, the safety of biostimulants is a concern, since it stipulates that they should only be placed on the market if they are sufficiently effective and do not present a risk to human beings, animals or plants health, safety or the environment, when properly stored and used for the purpose for which they are intended. Thus, the presence of contaminants such as *E. coli*, *Enterobactereacea*, and *Salmonella* sp. is limited.

For microbial biostimulants, the regulation currently permits the use of only four specific microorganisms: *Azotobacter* spp., *Rhizobium* spp., and *Azospirillum* spp., and *mycorrhizal fungi*. This limited list excludes many microorganisms already employed by companies in developing biostimulants, highlighting a gap in the regulation. However, there are expectations that this list will be expanded based on ongoing work by European groups (Ibáñez et al. 2023).

The practical benefits of having a well-defined regulatory framework are to favor the adoption of the technology by growers, giving greater certainty of the agronomic efficacy of the strains used due to the recommended tests. It also encourages research and strengthens the biofertilizer market, since everyone must to follow the same rules for production. In addition, by regulating packaging, labeling, and disposal, it provides a better understanding of product use and application.

### Biofertilizer market in the USA

**Fig. 1:**
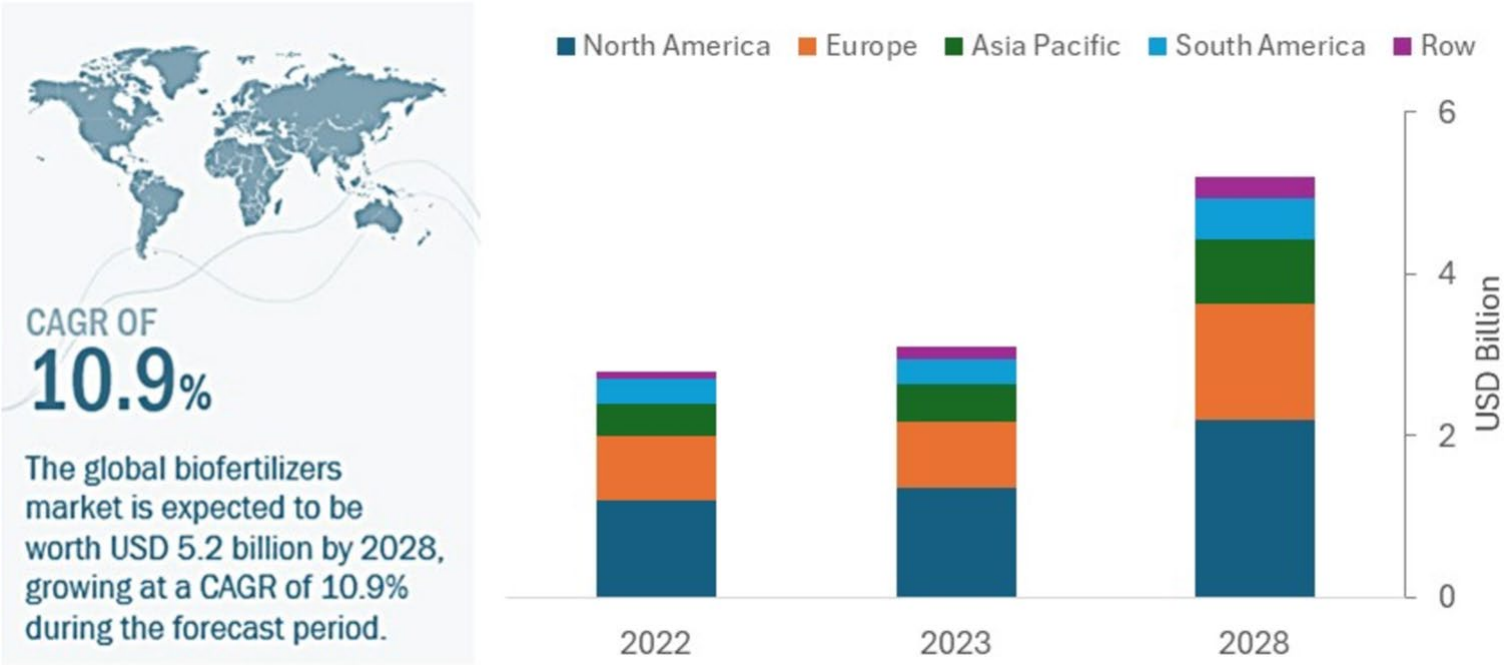
Biofertilizers Market Global Forecast to 2028 (USD BN) Source: adapted from MarketsandMarkets (2023)

**Fig. 2:**
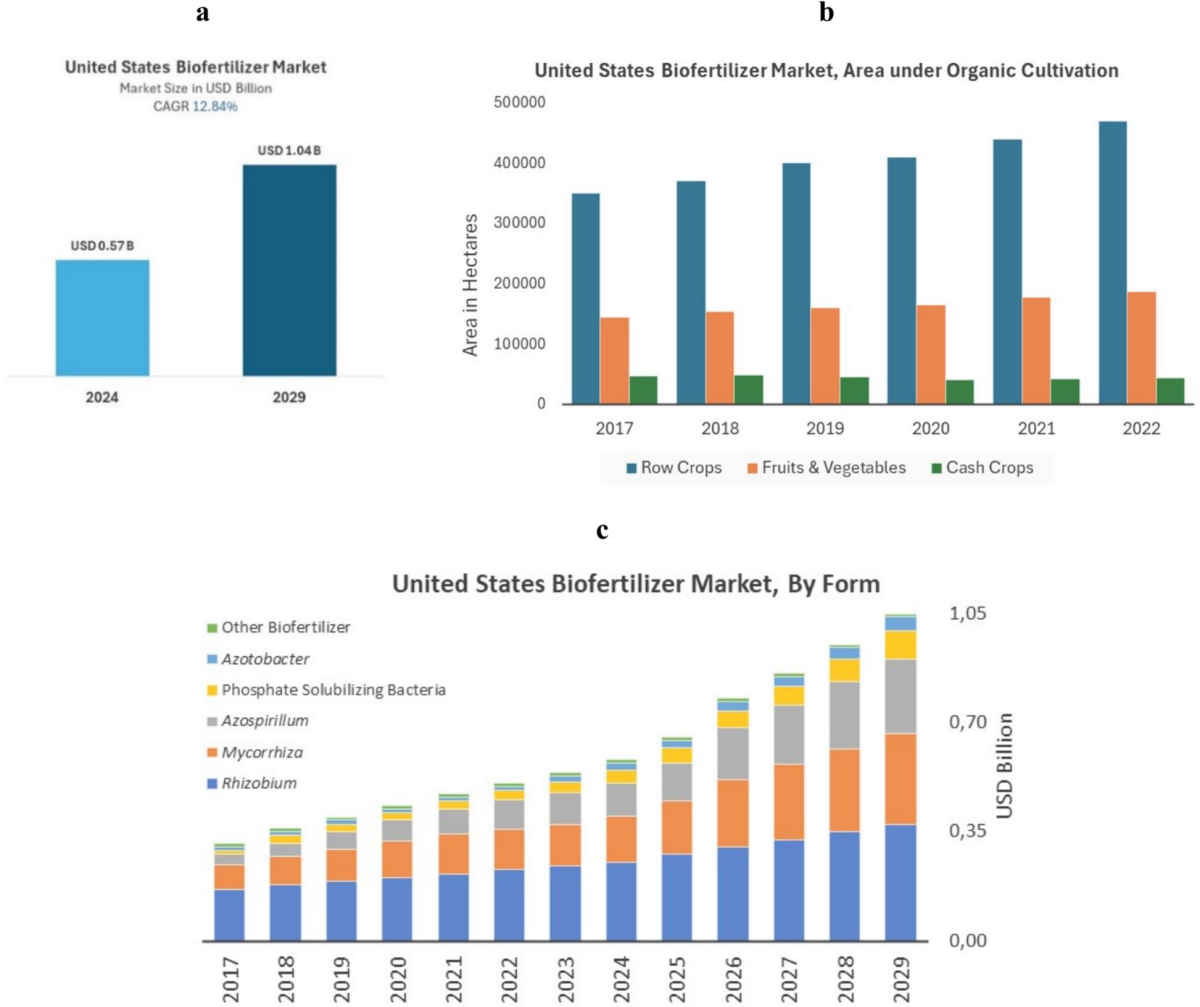
The USA Biofertilizer Market Size (**a**), USA Biofertilizer Market aera under organic cultivation (**b**), and USA Market by Form (**c**) Source: adapted from Mordor Intelligence (2024)

The biofertilizer inputs sector has been growing sharply around the world (CAGR of 10.9% 2023–2028) (Fig. 1) (MarktsandMarkets 2023) and also in the USA with a CAGR (Compound Annual Growth Rate) higher than the global one (12.84%) (Fig. 2a) (Mordor Intelligence 2024), stimulated by the growing demand for food production and new markets in Asia Pacific and Africa, the more demanding market for more sustainable agricultural production and climate change.

In terms of revenues generated from biofertilizer production, North America dominates the global biofertilizer market, followed by Europe (Germany, UK, Spain, Italy, Hungary, and France) and the Asia–Pacific region (China, Japan, India, Australia, New Zealand, and the rest of Asia), South America (Fig. 1), and Africa (Basu et al. 2021).

The growth of the biofertilizer market is also stimulated by the growth of organic farming (Mitter et al. 2021a, b; Newswire 2021; Yadav and Yadav 2024) and a large part of the products are consumed by row crops and fruits and vegetables (Fig. 2b).

Considering the form, or ingredients, the main biofertilizer products are composed of *Rhizobium*, followed by *Mycorrhizae*, *Azospirillum*, and phosphate solubilizing bacteria, and a smaller part of *Azobacteria* (Fig. 2c).

According to research carried out by Sansinenea (2021), Bayer Crop Science AG is a leading biofertilizer company in the world and acquired Monsanto BioAg, the biggest biofertilizer industry in Germany in 2018. The leaders of the US biofertilizers market are Kula Bio Inc., Novozymes, Rizobacter, Sustane Natural Fertilizer Inc., Symborg Inc., AgroLiquid, Indogulf BioAg LLC, Koppert Biological Systems Inc., Lallemand Inc., The Andersons Inc., UPL, Syngenta (Sansinenea 2021; MarketsandMarkets 2023; Mordor Intelligence 2024).

A study focused on how the importance of using biological inputs has grown elevated the voice of the farmers about this theme, released in April 2023 by the Stratovation Group that was commissioned by the Agricultural Retailers Association (ARA), Fertilizer Institute, and DCLRS (D.C. Legislative and Regulatory Services, Inc.), a bipartisan government relations firm (Eckelkamp 2023). The study was done in two phases: 40-h-long interviews and then a survey of 500 respondents—and both study groups were USA row crop farmers. The main insights of the study were that 83% of farmers in the US are aware of the term agricultural biologicals; the interest in the use of biological inputs has arisen due to the rise in fertilizer prices in recent years, and the availability of products in crop chemistry and the most important measure for success of biologicals were yield and profitability. Also, farmers’ willingness to try something new, reduce cost, increase N efficiency and decrease fertilizer expenses; on a scale of 0–10, with 0 being “not at all” and 10 being “very”, how interested are you in biological inputs?, farmers showed 6.43 to biofertilizers, 6.26 to biostimulants and 5.91 to biopesticides. Of those farmers not currently using biologicals, most said they would be willing to try them if their profitability could be proven. The survey shows that farmers need more details about the products and more education to help them understand the nuances of those products and associated with short shelf life, lack of proper storage, customer illiteracy, low consumer awareness, poor consumer guidelines, and insufficient production/promotion effort (Rai et al. 2023), which could explain the lack of widespread acceptance among growers in the USA.

### Biofertilizer database in the USA

**Fig. 3:**
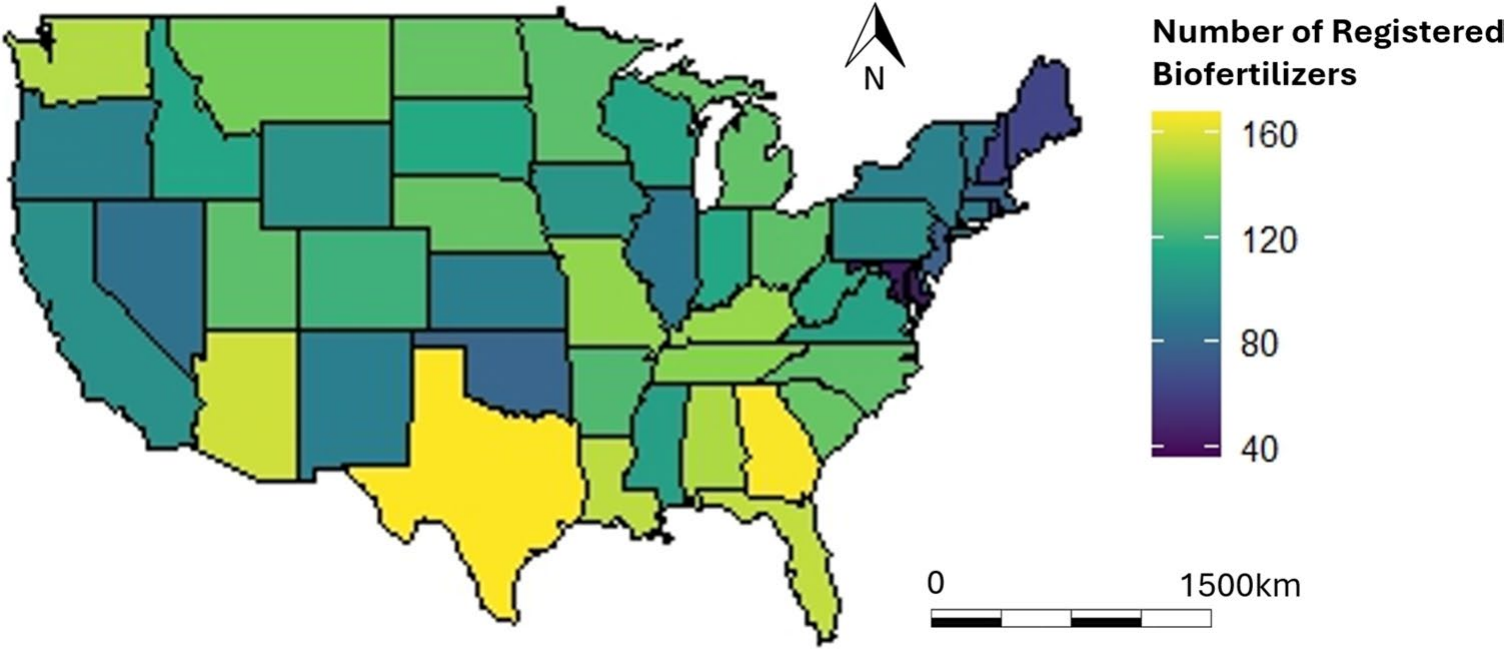
Number of biofertilizer registered by states in the USA

The database about the biofertilizer market in the USA shows the number of biofertilizers approved for use in each state in the USA (Fig. 3). Texas has the highest number of registered biofertilizers (167), followed closely by Georgia (166). Other important states in national agriculture, such as Arizona (157), Florida (154), Louisiana (153), Washington (151), and Alabama (151), also show large numbers of registered biofertilizers. Some states in the northeast and west have much lower number of registered biofertilizers. In future studies, maps such as this could be improved/updated by carefully delineating which registrations are “biofertilizers” according to a consensus definition, and which may fall in another category.

**Fig. 4:**
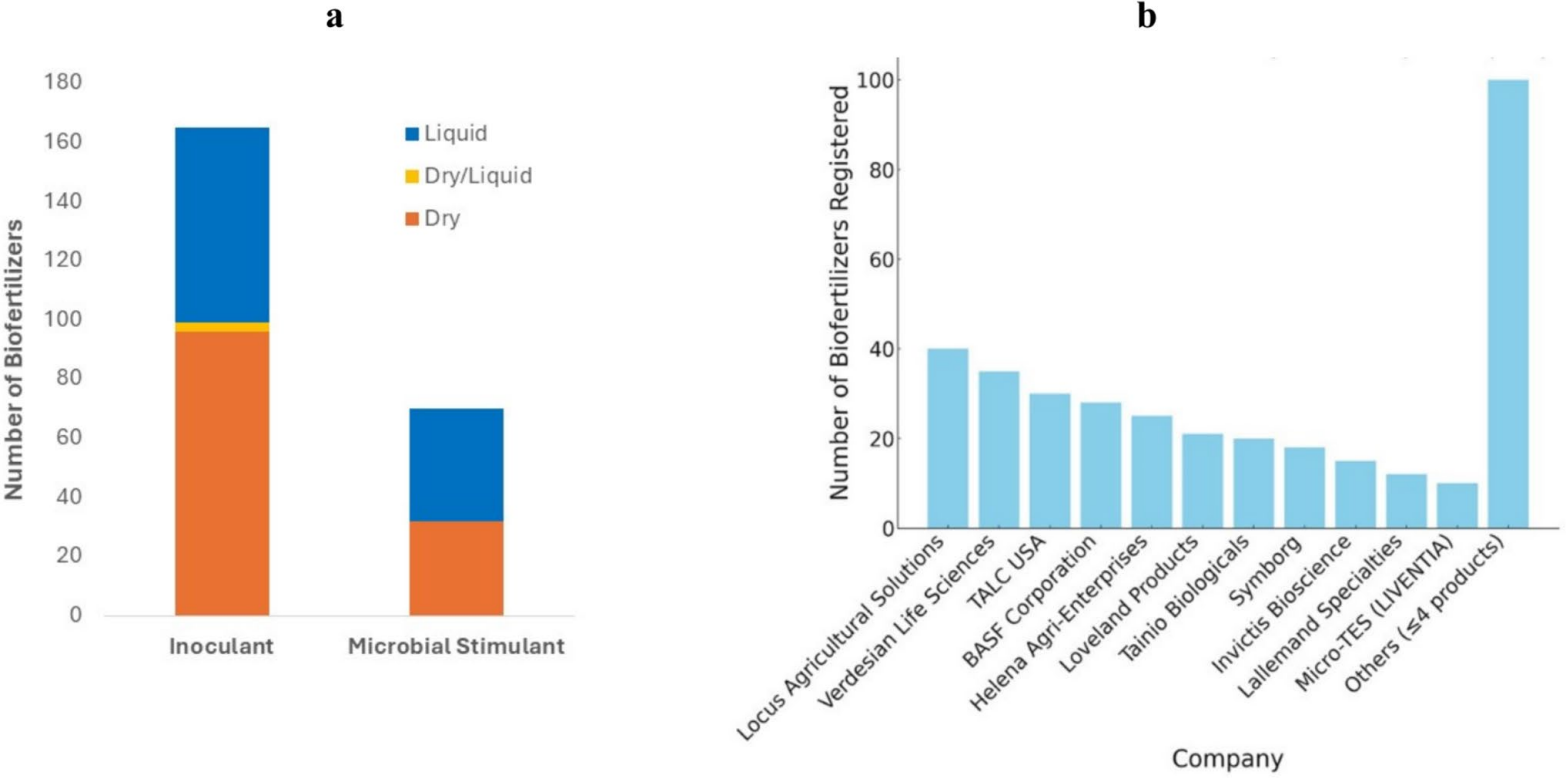
Number of physical states of the biofertilizers (**a**) and number of biofertilizers market by registrants (**b**)

In the inoculant group, the most common physical state of the products is solid, while in the microbial stimulants group, the liquid form of the products predominates (Fig. 4a). Some studies report that granules are superior to peat inoculants applied by seed, while others report the opposite (Clayton et al. 2004; Denton et al. 2009). Liquid formulations for in-furrow treatment are also popular with farmers as they are easy to handle, but although generally effective (Afzal et al. 2012, 2013), these types of inoculants may not provide protection against unfavorable soil conditions, as is the case with peat inoculants, and can be washed away by irrigation water or heavy rain.

Locus Agricultural Solutions and Verdesian Life Sciences are the registrants with the largest number of biofertilizers. However, there is a wide variety of registrants on the market, with most of the products divided among several different ones, represented in the graph as Others (Fig. 4b).

**Fig. 5:**
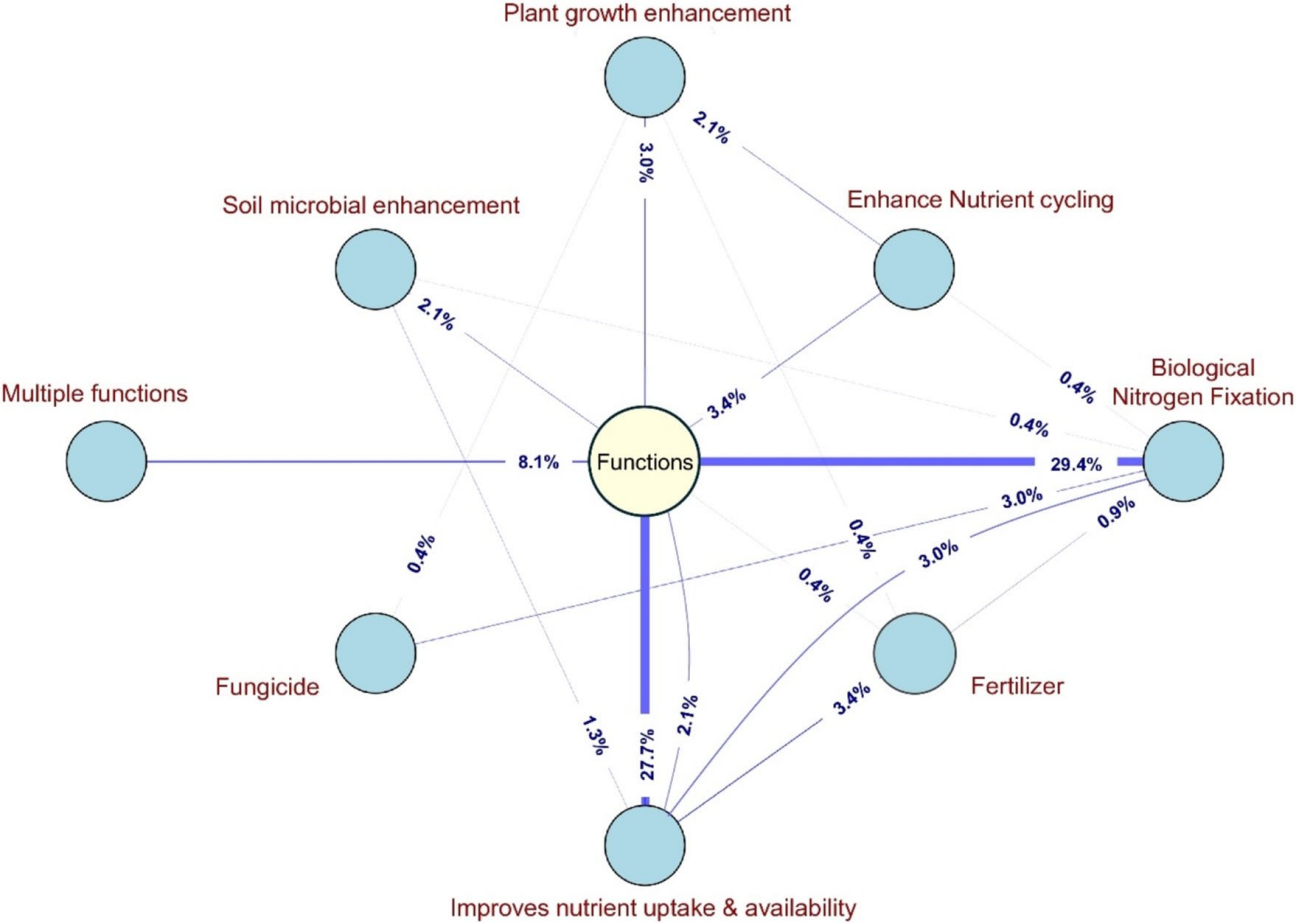
Functional interrelationships network diagram of biofertilizers in USA agriculture

Regarding the mechanism of action/function, according to the product labels, the network graph (Fig. 5) illustrates the various functions of biofertilizers used in the USA. The central yellow circle labeled “Functions” serves as a hub connecting the different specific functional categories of biofertilizers, represented by the surrounding blue circles. Single-function biofertilizers are represented by direct lines connecting the central circle to the outer blue circles. Dual-function biofertilizers are depicted by lines linking two blue circles. Biofertilizers with more than two functions fall into the multifunctional category, shown separately. The thickness of each line indicates the percentage of biofertilizers that serve each function, with thicker lines representing a higher percentage.

The majority of biofertilizers functions is for biological nitrogen fixation (29.4%) (Fig. 5) and confirms that the biofertilizer market continues to be dominated by nitrogen-fixing microorganisms, given that this nutrient is essential for plants and is demanded in greater quantities due to the long-term success of the rhizobium-legume symbiosis. Their dominance in the market is based on several factors, including well-demonstrated efficacy effectiveness in the field, research and development of standardized production and application approaches, and perhaps also the highly specific nature of the microbe-plant symbiosis (O’Callaghan et al. 2022).

As illustrated in Fig. 5, another important function demonstrated in this analysis is improvement of nutrient uptake and availability (27.7%) and the multiple functions represent 8.1% of the biofertilizers. However, many biofertilizers have dual functions, such as enhancing nutrient cycling (3.4%) and plant growth enhancement (2.1%) and soil microbial enhancement (2.1%), and improve nutrient uptake and availability (1.3%), as examples.

**Fig. 6:**
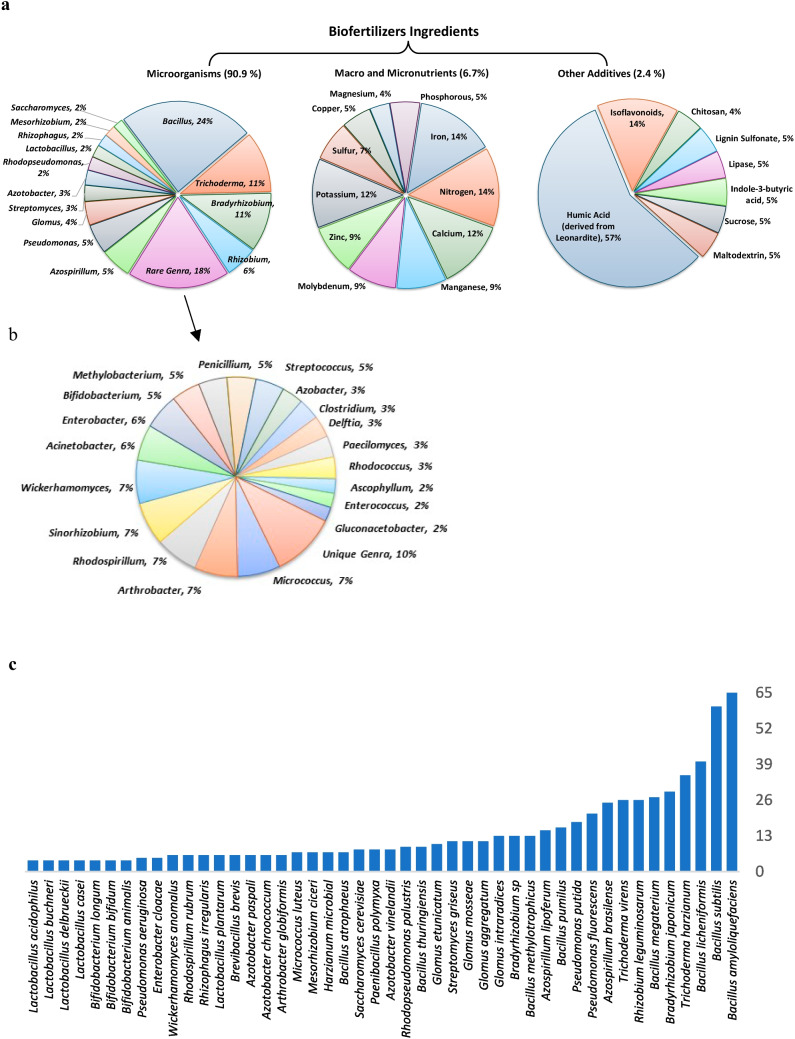
Percentage of biofertilizers ingredients and of microorganisms by genus (**a**), types of genus inside rare genera (**b**), and number of microorganisms ingredients by species in biofertilizers within USA (**c**)

Considering the ingredients (Fig. 6) more than 90% of the biofertilizers contain only microorganisms, and a small proportion of them also include nutrients (6.7%), such as N, Ca, and Fe, and other additives (2.4%)—mainly humic acid. Considering the microorganisms, the *Bacillus* genus is present in 24% of the biofertilizers analyzed; i.e., it is the most common genus in the products, followed by *Bradyrhizobium* (11%), *Trichoderma* (11%), *Rhizobium* (6%), *Azospirillum* (5%), and *Pseudomonas* (5%). The prevalence of these genera confirms the predominance of the functions of biological N fixation and improve nutrient uptake and availability, both of which are important uses for these types of microorganisms. The rare group accounts for 18% of biofertilizers and is well fragmented into different types of microorganisms, with a predominance of the genera *Micrococcus*, *Arthrobacter*, *Rhodospirillum*, *Sinorhizobium*, and *Wickerhamomyces*, each one with a 7% share (Fig. 6b).

Among the PGPR, the *Bacillus* genus is recognized as the most important and studied group of *bacteria* used in agricultural systems as biofertilizers or antagonists to many phytopathogens on different crops (Radhakrishnan et al. 2017). It is a Gram-positive, rod-shaped *bacterium* that can produce spores and has strong heat and stress resistance. It is commonly found in soil and on plant surfaces. This genus includes about 400 species with diverse physiological, metabolic, and genetic characteristics, and it holds significant biotechnological importance (Parte et al. 2020). For example, *Bacillus megaterium* species is named “the big beast” due to its large cellular size and its significant role in biotechnology for producing enzymes, antibiotics, hormones, and more (Bunk et al. 2010). In agriculture practices, many species of *Bacillus* (*B. subtilis*, *B. cereus*, *B. fusiformis*, *B. thuringiensis*, *B. pumilus*, *B. polymyxa*, *B. megaterium*, and *B. amyloliquefaciens*) are widely utilized to promote plant growth through multifunctional mechanisms such as phosphate solubilization, biological control, and the production of hormones, enzymes, and antibiotics (Aloo et al. 2019; Oliveira-Paiva et al. 2024). Within each genus, considering the species, *B. amyloliquefaciens* is the most widely used in biofertilizers (more than 60 products), given this gram-positive bacteria’s characteristics of plant growth promotion and to improve soil nutrient availability (Luo et al. 2022), followed by *B. subtilis* and *B. licheniformis* (Fig. 6c). The species *B. subtilis* solubilizes calcium and iron phosphate, has a high production of gluconic, indole acetic acid (IAA-like) and phytase enzyme (Velloso et al. 2020; Oliveira-Paiva et al. 2024), being a very important and efficient microorganism for use as biofertilizer.

Also, *B. subtilis* plays a significant role in improving tolerance to biotic stresses from induction of disease resistance involves the expression of specific genes and hormones (Aloo et al. 2019). The importance of *Bacillus* species is demonstrated by the approximately 3,106 patent applications for inoculant use worldwide (1374 in the USA) in the year of 2023, according to the Derwent World Patent Index (Oliveira and Santos 2023).

**Fig. 7:**
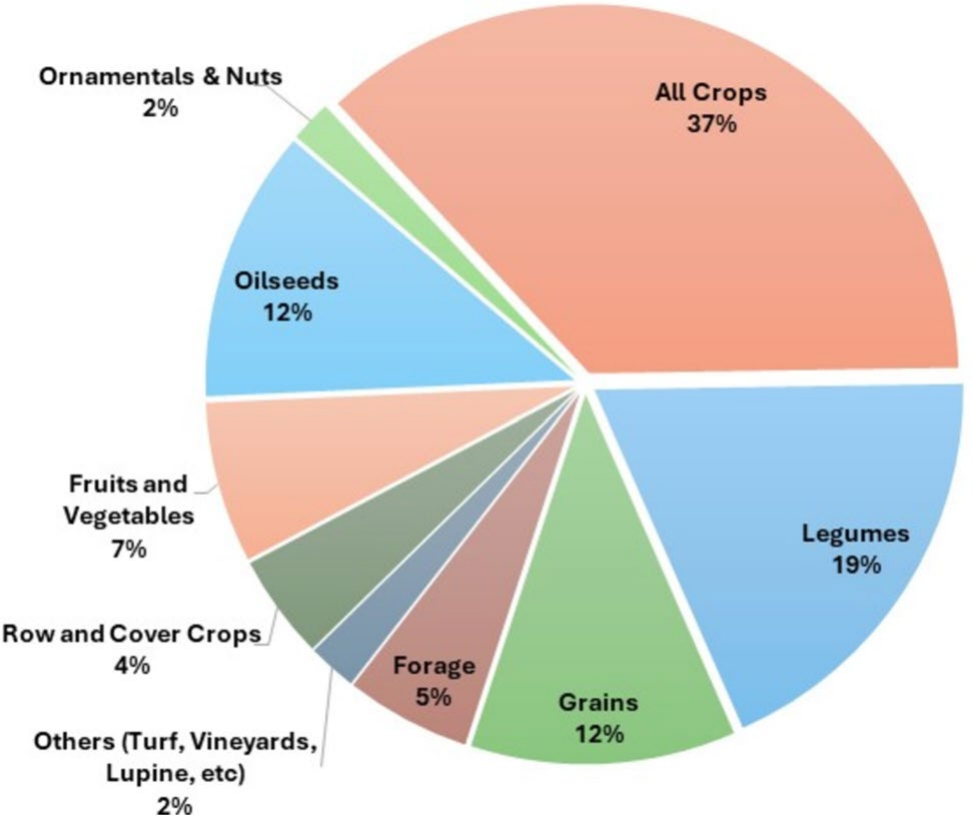
Biofertilizers used by crops within the USA

Most biofertilizers are used in all crops (37%), followed by legumes (19%), grains (12%), and oilseeds (12%) (Fig. 7). As many biofertilizers involve the function of biological N fixation, most crops are compound by legumes, in addition to a wider application, for all crops, as many biofertilizers have more than one function and even multiple functions (Fig. 5). The group of *Rhizobia* is known for its role in biological nitrogen fixation in leguminous plants. This group includes many genera, such as *Agrobacterium*, *Bradyrhizobium*, *Mesorhizobium*, *Rhizobium*, and *Sinorhizobium* (Olmo et al. 2022). By reducing atmospheric N2 through the nitrogenase enzyme complex, mineral nitrogen is produced and becomes available to plants. Seeds inoculated with *rhizobia* can promote plant development and grain production through multifunctional mechanisms, primarily biological N fixation (Olmo et al. 2022). The first biofertilizer formulated with *rhizobia*, called “Nitragin,” was developed in 1896. Recently, the genus *Bradyrhizobium* has been successful in improving soybean (*Glycine max* L.) grain yield and high capacity to biological N fixation, and in Brazil, for example, it is possible to obtain high yield without the need for mineral N fertilizers (Hungria and Mendes 2015). The successful program for using *Bradyrhizobium*-containing inoculants in Brazil allowed a considerable reduction in environmental degradation and production costs of around 79% for soybean, resulting in a saving of approximately US$ 15.2 billion in N fertilizers (Santos et al. 2021; Telles et al. 2023). Using *Rhizobia* biofertilizers in Zambia and the Republic of Congo also increased soybean yields by around 48% and 500 kg ha^−1^ (Raimi et al. 2021). Many countries have shown success in using *rhizobia*, which continues to stimulate new possibilities of use, as approximately 764 patent applications for *Rhizobia* were filed worldwide in 2023 (Oliveira and Santos 2023).

## Prospects

The constant demand for more sustainable agriculture, as well as the concern about the overuse of chemical fertilizers, support very promising prospects for biofertilizers, as the bio input sector is the fastest growing in agricultural activity and it has led to the emergence of a widely discussed term in the world today, which is the bioeconomy. The bioeconomy is defined as a model of industrial production based on the use of biological resources to guarantee sustainable development and the preservation of biodiversity (Oliveira and Santos 2023).

In the USA, as in the rest of the world, bio-inputs are mainly used in organic or agro-ecological agriculture, as they are fundamental to the management of these systems, but currently, the use of biological products is growing and becoming increasingly important in conventional agriculture, as an alternative or complement to fertilizers and phytosanitary products and to reduce production costs (Gindri et al. 2020), and this is an opportunity to expand markets.

The biggest market for biofertilizers is in America and USA had 2264 patent applications in 2023, of which 1558 were also filed in other countries. It is interesting to note that in the USA, companies are the main applicants, while in other countries, such as Brazil, there is more filing activity by government institutions and educational and research institutions (Oliveira and Santos 2023). This data shows a gap in the development of products by the government or public system and their opportunity to take up space.

The consortium of *bacteria* or *fungi* could be the new frontier in sustainable agriculture and there are research results confirm that with the use of formulation with multiple microorganisms the plants can produce more, increase nutrient uptake, and its possibly mitigate abiotic plant stresses, such as exposure to heavy metals, water deficit, and soil salinization (Raklami et al. 2019; Magallon-Servin et al. 2020; Embrapa 2024).

On the other hand, although there is ample evidence in the literature to indicate that microbial communities often work cooperatively to perform specific functions (Hungria et al. 2015), it is generally unclear how the combinations of microorganisms assembled in the products were selected and whether they work in unison or are competitive. A major challenge is the selection of consortium members such that performance is optimized and quantifying the benefits derived from the use of selected combinations of well-characterized microorganisms has not often been carried out and published (O’Callaghan et al. 2022).

The survival of microorganisms during production, formulation, storage, transportation, and field application under varying environmental conditions remains a future challenge. Efforts have been made to increase the shelf life of microbial inoculants. Research is being conducted to develop new formulations that provide a better environment for microbial survival and physical protection. This will improve cell viability over a longer period. Technological advances, including nanotechnology and microbial delivery systems (e.g., seed coating), as well as genetic modification of microorganisms, have been used to enhance the effectiveness of inoculants (Velloso et al. 2023). A challenge of using these bacteria is their reduced ability to survive when applied directly to the field or during storage since they need to endure deleterious abiotic and biotic environmental factors. Therefore, it is important to create microbial formulations to protect the cells from these stresses. Encapsulated formulations are an effective approach to accomplish this, as the carrier can protect the cells (Lopes et al. 2023). The development of new formulations presents a challenge in practical microbiology as it is a slow process. While many microbial strains have been identified, only a few are commercially effective (Bashan et al. 2014). Furthermore, enhancements in formulations are crucial for the creation of high-quality advanced inoculants high-end, considering microbial strains, carriers, and ably to plant growth.

The use of molecular engineering and nanotechnology can significantly enhance biofertilizer development (Yadav and Yadav 2024). Molecular tools enable to identify and characterize genes involved in critical processes such as N fixation, phosphate solubilization, and production of growth-promoting substances. Molecular engineering techniques such as CRISPR can be used to modify the genomes of biofertilizer microbes (e.g., bacteria and fungi) to enhance their beneficial traits. For instance, genes responsible for N fixation, phosphate solubilization, or plant growth hormone production can be overexpressed or modified to improve efficacy. Also, molecular engineering can help design and optimize microbial consortia (communities of multiple microbial species) to work synergistically, enhancing overall biofertilizer effectiveness. Seed coating and biopreparation for PGPRs and encapsulation approaches for biofertilizer strains have shown good results as they can improve the quality of inoculant products and there is demand for further research and development in this area. (Qiu et al. 2019; Rocha et al. 2019; O'Callaghan et al. 2022).

But along with the promising future of biofertilizers, there are challenges to overcome, and continued R&D is necessary to address gaps in knowledge and improve biofertilizer formulations and application methods. The biofertilizer product-based technology needs to be researched profoundly and improved to elicit desired results and gain the trust of the farmers, the real stakeholders of agriculture (Basu et al. 2021). There is a need for a better understanding of the mechanisms involved in the microorganism-soil–plant relationship, while the performance of biofertilizers can vary significantly depending on soil type, climate conditions, and crop species. This makes it challenging to predict their efficacy across different agricultural settings.

There is a need of developing adequate standards and legal provisions to support the production and use of biofertilizers (i.e., formulated products containing microorganisms). These could be initially developed at the international level through the ISO Standards, updating the currently available standards for fertilizers and soil conditioners (Malusá and Vassilev 2014). Microbial products need to highlight the application of some minimum requirements around concentration (CFU/ml or CFU/g) and also around eliminating or minimizing contaminants to perform well in the field. The implementation of microbiological and/or molecular methods is essential to confirm the presence of each strain and quantify its guaranteed number of cells. Therefore, Malusá et al. (2017) state that international standards could update and equalize the standards available for biofertilizers, following the example of ISO Standard 7851:1983 (Fertilizers and soil conditioners) and ISO Standard 8157:1984 (Fertilizers and soil conditioners).

Well-defined standards are important, since many stages of research and development are involved in the production of a biofertilizer. The stages of isolation, taxonomic identification, and the ability of microorganisms to promote plant growth through various mechanisms require time and investment. With the growth of the biological products market in general, it is essential to regularize procedures aimed at maintaining the safety of commercialized products. In this sense, the taxonomic identification of microbial strains, genetic purity, and maintenance of physiological processes of interest need to come from research bodies and official collections, with total security and supervision by public bodies. In this case, the production line for bio-inputs must have strict quality control stages to avoid contamination by disease-causing agents or the proliferation of species that produce toxins harmful to human health. This information is important, since in recent times the idea of producing bio-inputs has gone beyond the boundaries of industrial parks, and the construction of bio-factories on rural properties is being encouraged.

This practice is known as on-farm production and is aimed at producing bio-inputs for own consumption. It is often practiced through the non-recommended multiplication of commercial products purchased on the market on farms, with the aim of reducing production costs. In other words, in the on-farm system there is doubt as to whether what is being cultivated is the microorganisms of interest or whether there are contaminants present (e.g., fecal and total coliforms). Well-established production practices defined by accessible regulations provide the agricultural market with security in terms of the food safety of products, avoiding possible sanitary barriers due to the biological risk resulting from lax agricultural practices. Although the classifications of the microorganisms that formulate biofertilizers are well-defined scientifically, their practical application still presents problems in the legal sphere and there is a need to develop standards and legal provisions to support the production and use of biofertilizers (Ammar et al. 2023). Majority of plant growth promoting microorganism are classified as biosafety level 1, meaning they are not known to cause disease (Shepherd et al. 2010), but laboratories must check the level of pathogenicity or other associated danger with microorganisms (Rai et al. 2023). Furthermore, exotic microbes can affect the functioning of the soil microbiome by causing invasive and competitive reactions (Sathya et al. 2017).

The market demands a federal standard to overcome the state differences in regulatory pathways so a single product can be sold nationwide. Overall, federal/nationwide standards enable nationwide commerce with a single label versus a multitude of state requirements (Caldwell and Fife 2023). All these issues being addressed will be very important for growers to be able to separate what is a quality product with proven efficacy or not.

## Supplementary information

Below is the link to the electronic supplementary material.

Supplementary file1 (PDF 796 KB)

## Data Availability

The authors declare that the data supporting the findings of this study are available within the paper and its Supplementary Information files. The data about Biofertilizer market in the United States can be found in MarktsandMarkets (2023) in this link https://www.marketsandmarkets.com/Market-Reports/compound-biofertilizers-customized-fertilizers-market-856.html) and Mordor Intelligence (2024) in this link https://www.mordorintelligence.com/industry-reports/north-america-biofertilizer-market. All other relevant data generated and analyzed during this study, which include biofertilizer database in the USA, are provided in this link https://agrian.com/labelcenter/results.cfm?s = .

## References

[CR1] Afzal M, Yousaf S, Reichenauer TG, Sessitsch A (2012) The inoculation method affects colonization and performance of bacterial inoculant strains in the phytoremediation of soil contaminated with diesel oil. Int J Phytor 14:35–47. 10.1016/j.ibiod.2013.08.022 10.1080/15226514.2011.552928 22567693

[CR2] Afzal M, Khan S, Iqbal S, Mirza MS, Khan QM (2013) Inoculation method affects colonization and activity of *Burkholderia phytofirmans* PsJN during phytoremediation of diesel-contaminated soil. Int Biodeter Biodegrad 85:331–336. 10.1080/15226514.2011.552928

[CR3] Aloo BN, Makumba BA, Mbega ER (2019) The potential of *Bacilli rhizobacteria* for sustainable crop production and environmental sustainability. Microbiol Res 219:26–39. 10.1016/j.micres.2018.10.011 30642464 10.1016/j.micres.2018.10.011

[CR4] Aloo BN, Tripathi V, Makumba BA, Mbega ER (2022) *Plant growth-promoting rhizobacterial* biofertilizers for crop production: the past, present, and future. Front Plant Sci 13:1002448. 10.3389/fpls.2022.1002448 36186083 10.3389/fpls.2022.1002448 PMC9523260

[CR5] Ammar EE, Rady HA, Mustafa A, Amer MH, Mohamed SA, Elodamy NI, Al-Farga A, Aioub A (2023) A comprehensive overview of eco-friendly bio-fertilizers extracted from living organisms. Environ Sci Pollut Res Int 30:113119–113137. 10.1007/s11356-023-30260-x 37851256 10.1007/s11356-023-30260-x PMC10663222

[CR6] Backer R, Rokem JS, Ilangumaran G, Lamont J, Praslickova D, Ricci E, Subramanian S, Smith DL (2018) *Plant growth promoting rhizobacteria*: context, mechanisms of action, and roadmap to commercialization of biostimulants for sustainable agriculture. Front Plant Sci 9:1473 30405652 10.3389/fpls.2018.01473 PMC6206271

[CR8] Bashan Y, de-Bashan LE, Prabhu SR, Hernandez JP (2014) Advances in *plant growth-promoting bacterial* inoculant technology: formulations and practical perspectives (1998–2013). Plant Soil 378:1–33. 10.1007/s11104-013-1956-x

[CR9] Basu A, Prasad P, Das SN, Kalam S, Sauuco RZ, Reddy MS, El Elenshasy H (2021) *Plant Growth Promoting Rhizobacteria* (PGPR) as green bioinoculants: recent developments, constraints, and prospects. Sustainability 13:1140. 10.3390/su13031140

[CR10] Biological Products Industry Alliance - BPIA (2023) The listening session report: identifying ambiguities, gaps, inefficiencies, and uncertainties in the coordinated framework for the regulation of biotechnology. p 27

[CR11] Bunk B, Schulz A, Stammen S, Münch R, Warren MJ, Rohde M, Jahn D, Biedendieck R (2010) A short story about a big magic bug. Bioengineered Bugs 1(2):85–91. 10.4161/bbug.1.2.11101 21326933 10.4161/bbug.1.2.11101 PMC3026448

[CR12] Caldwell B, Fife J (2023) Biologicals regulation: the right way. https://www.3barbiologics.com/2023/07/biologicals-regulation-the-right-way/#:~:text=The%20regulatory%20process%20is%20divided,microorganism%20is%20the%20active%20ingredient. Acessed 15 May 2024

[CR13] Chaudhary P, Singh S, Chaudhary A, Sharma A, Kumar G (2022) Overview of biofertilizers in crop production and stress management for sustainable agriculture. Front Plant Sci 13:930340 36082294 10.3389/fpls.2022.930340 PMC9445558

[CR14] Clayton GW, Rice WA, Lupwayi NZ, Johnston AM, Lafond GP, Grant CA, Walley F (2004) Inoculant formulation and fertilizer nitrogen effects on field pea: nodulation, N2 fixation and nitrogen partitioning. Can J Plant Sci 84:79–88. 10.4141/P02-089

[CR15] Daniel AI, Fadaka AO, Gokul A, Bakare OO (2022) Biofertilizer: the future of food security and food safety. Microorganisms 10:1120. 10.3390/microorganisms10061220 35744738 10.3390/microorganisms10061220 PMC9227430

[CR16] Denton MD, Pearce DJ, Ballard RA, Hannah MC, Mutch LA, Norng S, Slattery JF (2009) A multi-site field evaluation of granular inoculants for legume nodulation. Soil Biol and Biochem 41:2508–2516. 10.1016/j.soilbio.2009.09.009

[CR17] Dineshkumar R, Kumaravel R, Gopalsamy J, Sikder MNA, Sampathkumar P (2018) *Microalgae* as bio-fertilizers for rice growth and seed yield productivity. Waste Biom Valoriz 9:793–800

[CR18] du Jardin P (2015) Plant biostimulants: definition, concept, main categories, and regulation. Scientia Horticul 196:3–14. 10.1016/j.scienta.2015.09.021

[CR19] Dunham WC (2015) Evolution and future of biocontrol. Dunham Trimmer International Bio Intelligence. TwoB Monthly – The Global Biocontrol &b Biostimulants E-Newsletter, p 23

[CR20] Eckelkamp M (2023) Biologicals: how Ag retailers can be a springboard for growth. New Products, The Scoop. https://www.thedailyscoop.com/news/new-products/biologicals-how-ag-retailers-can-be-springboard-growth. Acessed 16 May 2024

[CR21] Embrapa (2024) Agencia Embrapa de Noticias. https://www.embrapa.br/busca-de-noticias/-/noticia/89417452/uso-de-grupos-de-microrganismos-e-a-nova-fronteira-em-bioinsumos). Acessed 16 May 2024

[CR22] EPA (2020) Draft guidance for plant regulator products and claims, including plant biostimulants. https://www.epa.gov/sites/default/files/2020-11/documents/pbs-guidance-updated-draft-guidance-document-2020-11-13_0.pdf. Acessed 15 May 2024

[CR23] Fasusi OA, Cruz C, Babalola OO (2021) Agricultural sustainability: microbial biofertilizers in rhizosphere management. Agriculture 11:163. 10.3390/agriculture11020163

[CR24] Gindri D, Moreira PAB, Veríssimo MAA (2020) Sanidade vegetal: uma estratégia global para eliminar a fome, reduzir a pobreza, proteger o meio ambiente e estimular o desenvolvimento econômico sustentável

[CR25] Hungria M, Mendes IC (2015) Nitrogen fixation with soybean: the perfect symbiosis? In: de Bruijn FJ (ed) Biological Nitrogen Fixation. Wiley, Hoboken, New Jersey, pp. 1009–1023. 10.1002/9781119053095.ch99.

[CR26] Hungria M, Nogueira MA, Araujo RS (2015) Soybean seed co-inoculation with *Bradyrhizobium spp* and *Azospirillum brasilense*: a new biotechnological tool to improve yield and sustainability. Am J Plant Sci 6:811–817. 10.4236/ajps.2015.66087

[CR27] Ibáñez A, Garrido-Chamorro S, Vasco-Cárdenas MF, Barreiro C (2023) From lab to field: biofertilizers in the 21st century. Horticulturae 9:1306. 10.3390/horticulturae9121306

[CR28] Kloepper JW (1993) *Plant-growth-promoting rhizobacteria* as biological control agents. In: Metting EB (ed) Soil microbial ecology: applications in agricultural and environmental management. Marcel Dekker Inc., New York, pp 255–273

[CR29] Kumar S, Sindhu SS, Kumar R (2022) Biofertilizers: an ecofriendly technology for nutrient recycling and environmental sustainability. Cur Res Microbial Sci 20:100094. 10.1016/j.crmicr.2021.100094 10.1016/j.crmicr.2021.100094 PMC8724949 35024641

[CR30] Lopes MM, Oliveira-Paiva CA, Farinas CS (2023) Modification of pectin/starch-based beads with additives to improve *Bacillus subtilis* encapsulation for agricultural applications. Int J Biol Macrom 246:125646. 10.1016/j.ijbiomac.2023.125646 10.1016/j.ijbiomac.2023.125646 37394222

[CR31] Luo L, Zhao C, Wang E, Raza A, Yin C (2022) *Bacillus amyloliquefaciens* as an excellent agent for biofertilizer and biocontrol in agriculture: an overview for its mechanisms. Microbial Res 259:127016 10.1016/j.micres.2022.127016 35390741

[CR32] Magallon-Servin P, Antoun H, Taktek S, De-Bashan LE (2020) Designing a multi-species inoculant of *phosphate rock-solubilizing bacteria* compatible with *arbuscular mycorrhizae* for plant growth promotion in low-P soil amended with PR. Biol Fertil Soils 56:521–536. 10.1007/s00374-020-01452-1

[CR33] Maitra S, Brestic M, Bhadra P, Shankar T, Praharaj S, Palai JB, Shah MMR, Barek V, Ondrisik P, Skalický M, Hossain A (2021) Bioinoculants-natural biological resources for sustainable plant production. Microorganisms 10(1):51. 10.3390/microorganisms10010051 35056500 10.3390/microorganisms10010051 PMC8780112

[CR34] Malusá E, Vassilev N (2014) A contribution to set a legal framework for biofertilisers – mini-review. Appl Microbiol Biotechnol 98:6599–6607. 10.1007/s00253-014-5858-y 24903811 10.1007/s00253-014-5828-y PMC4108841

[CR35] Malusá E, Canfora L, Pinzari F, Tartanus M, Tabanowska BH (2017) Improvement of soilborne pests control with agronomical pratices exploiting the interaction of *Entomaphagous fungi*. In: Singh D, Singh H, Prabha R (eds) Plant Perspectives Springer: Singapore. 10.1007/978-981-10-5813-4-29

[CR36] MarketsandMarkets (2023) Biofertilizers Market – Report Code AGI 2569. https://www.marketsandmarkets.com/Market-Reports/compound-biofertilizers-customized-fertilizers-market-856.html. Acessed 16 May 2024

[CR37] Mercier S (2023) The use of “biologicals in agriculture. Opinion, Fram Journal AG WEB. https://www.agweb.com/opinion/use-biologicals-agriculture. Acessed 15 May 2024

[CR38] Mitter EK, Tosi M, Obreg´on D, Dunfield KE, Germida JJ (2021) Rethinking crop nutrition in times of modern microbiology: innovative biofertilizer technologies. Front Sustain Food Syst 5:606815. 10.3389/fsufs.2021.606815

[CR39] Mitter EK, Tosi M, Obregón D, Dunfield KE, Germida JJ (2021b) Rethinking crop nutrition in times of modern microbiology: innovative biofertilizer technologies. Front Sustain Food Syst 5:606815. 10.3389/fsufs.2021.606815

[CR40] Mordor Intelligence (2024) United States Biofertilizer Market size & share analysis -Growth trends & forecasts up to 2029. https://www.mordorintelligence.com/industry-reports/north-america-biofertilizer-market. Acessed 15 May 2024

[CR41] Newswire (2021) Global organic food market report (2021 to 2030) - featuring general Mills, Cargill and Danone among others. https://www.globenewswire.com/news-release/2021/11/23/2339637/28124/en/Global -Organic-Food-Market-Report-2021-to-2030-Featuring-General-Mills-Cargill -and-Danone-Among-Others.html. Acessed 16 May 2024

[CR42] Nosheen S, Ajmal I, Song Y (2021) Microbes as biofertilizers, a potential approach for sustainable crop production. Sustainability 13:1868

[CR43] O’Callaghan M, Ballard R, Wright D (2022) Soil microbial inoculants for sustainable agriculture: Limitations and opportunities. Soil Use Manage 38:1340–1369

[CR44] Okon Y, Labandera-Gonzalez CA (1994) Agronomic applications of *Azospirillum*: an evaluation of 20 years worldwide field inoculation. Soil Biol Biochem 26:1591–1601

[CR45] Oliveira SS, Santos PR (2023) Bioinsumos na agricultura: inoculantes. Rio de Janeiro: Instituto Nacional da Propriedade Industrial (Brasil) – INPI, Diretoria de Patentes, Programas de Computador e Topografia de Circuitos Integrados - DIRPA, Coordenação Geral de Estudos, Projetos e Disseminação da Informação Tecnológica - CEPIT e Divisão de Estudos e Projetos – DIESP, 2023. p 62

[CR46] Oliveira-Paiva CA, Bini D, De Sousa SM, Ribeiro VP, Dos Santos FC, Gomes U, De Souza FF, Gomes EA, Marriel IE (2024) Inoculation with *Bacillus megaterium* CNPMS B119 and *Bacillus subtilis* CNPMS B2084 improve P-acquisition and maize yield in Brazil. Front in Microbiol 15:1426166. 10.3389/fmicb.2024.1426166 10.3389/fmicb.2024.1426166 PMC11233657 38989019

[CR47] Olmo R, Wetzels SU, Armanhi JS, Arruda P, Berg G, Cernava T, Cotter PD, Araujo SC, Souza RSC, Ferrocino I, Frisvad JC, Georgalaki M, Hansen HH, Kazou M, Kiran GS, Kosteg T, Krausc-Etschman S, Kniaa A, Lange L, Maguin E, Mitter B, Nielsen MO, Olivares M, Quijada NM, Romani-Perez M, Sanz Y, Schloter M, Schmitt-Kopplin P, Seaton SC, Servin J, Sessitsch A, Wang M, Zwirzit B, Selbonherr E, Wagner M (2022) Microbiome research as an effective driver of success stories in agrifood systems – a selection of case studies. Front Microbiol 13:834622. 10.3389/fmicb.2022.834622 35903477 10.3389/fmicb.2022.834622 PMC9315449

[CR48] Parte AC, Sarda Carbasse J, Meier-Kolthoff JP, Reimer LC, Goker M (2020) List of *Prokaryotic* names with Standingin Nomenclature (LPSN) moves to the DSMZ. Int J Syst Evol Microbiol 70(11):5607–5612. 10.1099/ijsem.0.004332 32701423 10.1099/ijsem.0.004332 PMC7723251

[CR49] Patel C, Singh J, Karunakaran A, Ramakrishna W (2023) Evolution of nano-biofertilizer as a green technol agricult. Agriculture 13:1865

[CR51] Qiu Z, Egidi E, Liu H, Kaur S, Singh BK (2019) New frontiers in agriculture productivity: optimised microbial inoculants and in situ microbiome engineering. Biotechnol Adv 37:107371. 10.1016/j.biotechadv.2019.03.010 30890361 10.1016/j.biotechadv.2019.03.010

[CR52] Radhakrishnan R, Abeer H, Elsayed F (2017) Bacillus: a biological tool for crop improvement through bio-molecular changes in adverse environments. Front Physiol 8:293128. 10.3389/fphys.2017.00667 10.3389/fphys.2017.00667 PMC5592640 28932199

[CR53] Rai PK, Rai A, Sharma NK, Sing T, Kumar Y (2023) Limitations of biofertilizers and their revitalization through nanotechnology. J Cleaner Prod 418:138194

[CR54] Raimi A, Roopnarain A, Adeleke R (2021) Biofertilizer production in Africa: current status, factors impeding adoption and strategies for success. Sci Afr 11:e00694. 10.1016/j.sciaf.2021.e00694

[CR55] Raklami A, Bechtaoui N, Tahiri A, Anli M, Meddich A, Oufdou K (2019) Use of *rhizobacteria* and *mycorrhizae* consortium in the open field as a strategy for improving crop nutrition, productivity, and soil fertility. Front Microbiol 10:1–11 31164880 10.3389/fmicb.2019.01106 PMC6536659

[CR56] Ravensberg W (2017) The future of microbial products and regulatory issues. IBMA – International Biocontrol Manufacturers Association. https://ibma-global.org/wp-content/uploads/2020/12/ravensbergthefutureofmicrobialproductsandregulatoryissues.pdf. Acessed 15 May 2024

[CR57] Rehman A, Ma H, Ahmad M, Irfan M, Traore O, Chandio AA (2021) Towards environmental sustainability: devolving the influence of carbon dioxide emission to population growth, climate change, Forestry, livestock and crops production in Pakistan. Ecol Indicat 125:107460. 10.1016/j.ecolind.2021.107460

[CR58] Rocha IR, Ma Y, Souza-Alonso P, Vosatka M, Freita H, Oliveira RS (2019) Seed coating: a tool for delivering beneficial microbes to agricultural crops. Front Plant Sci 10:1357. 10.3389/fpls.2019.01357 31781135 10.3389/fpls.2019.01357 PMC6852281

[CR60] Sansinenea E (2021) Application of biofertilizers: current worldwide status. In: Biofertilizers. Woodhead Publishing, pp 183–190. 10.1016/B978-0-12–821667–5.00004-X

[CR61] Santos MS, Hungria M, Nogueira MA (2021) Outstanding impact of *Azospirillum brasilense* strains Ab-V5 and Ab-V6 on the Brazilian agriculture: lessons that farmers are receptive to adopt new microbial inoculants. Rev Bras Ci Solo 45:E200128. 10.36783/18069657rbcs20200128

[CR62] Sathya A, Vijayabharathi R, Gopalakrishnan S (2017) Plant growth-promoting actinobacteria: a new strategy for enhancing sustainable production and protection of grain legumes. 3 Biotech 7(2):1–10 10.1007/s13205-017-0736-3 PMC5449283 28560641

[CR63] Scope Newsletter - European sustainable phosphorus platform (2024) ESPP stakeholder workshop: Towards a definition of “Bio-Based” nutrients 150 pp 9–12

[CR64] Shahwar D, Mushtaq Z, Mushtaq H, Alqarawi AA, Park Y, Alshahrani TS, Faizan S (2023) Role of microbial inoculants as bio fertilizers for improving crop productivity: a review. Heliyon. 10.1016/j.heliyon.2023.e16134 37255980 10.1016/j.heliyon.2023.e16134 PMC10225898

[CR65] Sharma A, Chetani R (2017) A review on the effect of organic and chemical fertilizers on plants. IJRASET 5(2):677–680 (**ISSN: 2321-9653**)

[CR66] Shepherd MD, Kharel MK, Bosserman MA, Rohr J (2010) Laboratory maintenance of Streptomyces species. Curr Protocol Microbiol 18(1):10E. 11.11-10E. 11.18 10.1002/9780471729259.mc10e01s18 PMC2950629 20812215

[CR68] Singh V, Kumar B (2024) A review of agricultural microbial inoculants and their carriers in bioformulatio. Rhizsosphere 29:100843

[CR69] Somers E, Vanderleyden J, Srinivasan M (2004) Rhizosphere bacterial signaling: a love parade beneath our feet. Crit Rev Microbiol 30:205–240 15646398 10.1080/10408410490468786

[CR70] Stewart W, Roberts T (2012) Food security and the role of fertilizer in supporting it. Procedia Eng 46:76–82

[CR71] Telles TS, Nogueira MA, Hungria M (2023) Economic value of biological nitrogen fixation in soybean crops in Brazil. Environ Technol Innov 31:103158

[CR72] Telus Agronomy (2024) https://agrian.com/labelcenter/results.cfm?s=. Acessed 16 May 2024

[CR73] Velloso CC, Oliveira-Paiva CA, Gomes EA, Lana UGP, Carvalho CG, Guimaraes LJM, Pastina MM, Sousa SM (2020) Genome-guided insights of tropical *Bacillus* strains efficient in maize growth promotion. FEMS Microbiol Ecology 96(9):fiaa157. 10.1093/femsec/fiaa157 10.1093/femsec/fiaa157 32785605

[CR74] Velloso CC, Camargo BCP, Sousa MDB, Buffo MM, Oliveira-Paiva CA, Farinas CS, Badino AC (2023) High yield of heat-resistant spores of Bacillus megaterium in bioreactors. Biochem Eng J 198:109030. 10.1016/j.bej.2023.109030

[CR75] Vessey JK (2003) *Plant growth promoting rhizobacteria* as biofertilizers. Plant Soil 255:571–586

[CR76] Yadav KK, Sarkar S (2019) Biofertilizers, impact on soil fertility and crop productivity under sustainable agriculture. Environ Ecol 37:89–93

[CR77] Yadav A, Yadav K (2024) Challenges and opportunities in biofertilizer commercialization. SVOA Microbiol 5(1):1–14. 10.58624/SVOAMB.2024.05.037

