# Supplementary material for: Biofertilizer use in the United States: definition, regulation, and prospects
Source: Appl Microbiol Biotechnol. 2024 Nov 12;108:511. doi: 10.1007/s00253-024-13347-4 (PMC11557716; doi:10.1007/s00253-024-13347-4)
Supplement: Supplementary file 1 [file 253_2024_13347_MOESM1_ESM.pdf]

Supplementary information

Title Page

Applied Microbiology and Biotechnology

Mini-Review

Biofertilizer use in the United States: definition, regulation, and prospects

Flavia Santos
Soil, Water & Ecosystem Sciences Department, Everglades REC, University of Florida, 3200 E Canal St S., Belle Glade, FL, USA 33430, flaviasantos@ufl.edu
Embrapa Maize and Sorghum, Rod MG 424, Rod. MG 424 KM 65, CP 285 or 151, 35.701-970, Sete Lagoas, MG, Brazil, flavia.santos@embrapa.br

Suraj Melkani
Soil, Water & Ecosystem Sciences Department, Everglades REC, University of Florida, 3200 E Canal St S., Belle Glade, FL, USA 33430, suraj.melkani@ufl.edu

Christiane Oliveira-Paiva
Embrapa Maize and Sorghum, Rod MG 424, Rod. MG 424 KM 65, CP 285 or 151, 35.701-970, Sete Lagoas, MG, Brazil, christiane.paiva@embrapa.br

Daniel Bini
Embrapa Maize and Sorghum, Rod MG 424, Rod. MG 424 KM 65, CP 285 or 151, 35.701-970, Sete Lagoas, MG, Brazil, dbini.master@gmail.com

Kiran Pavuluri

Supplementary information

International Fertilizer Development Center - IFDC Headquarters, P.O. Box 2040, Muscle Shoals, AL, USA 35662, kpavuluri@ifdc.org

Luke Gatiboni
North Caroline State Extension, North Caroline State University, Campus Box 7619, Raleigh, NC 27695, luke_gatiboni@ncsu.edu

Anik Mahmud
Soil, Water & Ecosystem Sciences Department, Everglades REC, University of Florida, 3200 E Canal St S., Belle Glade, FL, USA 33430, md.mahmud@ufl.edu

Maria Torres
Plant and Environmental Sciences, Clemson University, 105 Sikes Hall, Clemson, SC, USA 29634, mtorre3@clemson.edu

Eric McLamore
Plant and Environmental Sciences, Clemson University, 105 Sikes Hall, Clemson, SC, USA 29634, emclamo@clemson.edu

Jehangir Bhadha
Soil, Water & Ecosystem Sciences Department, Everglades REC, University of Florida, 3200 E Canal St S., Belle Glade, FL, USA 33430, jango@ufl.edu, phone 561 993 1500, fax 561 993 1582 (corresponding author)

1. Syntactic analysis of definitions extracted from peer reviewed literature

Table S1 shows a concatentated list of the definitions from various sources. In summary:

- Eight different definitions of biofertilizer in the peer reviewed literature
- Four different definitions of biostimulant
- Three different definitions of biopesticide
- Bioinoculant and enhancer each had only one definition

Supplementary information

Table S1. Summary of key terms and formal definitions in the scientific literature. For brevity, terms from policy were not included in table.

**Term**	**Definition** (Source)	**Definition** (Source)	**Definition** (Source)
Biofertilizer	rhizospheric organism(s) capable of improving the use of soil nutrients, but not replacing them<br><br>(Okon and Labandera-Gonzalez 1994)	"…a substance which contains living microorganisms which when applied to seed, plant surfaces, or soil, colonizes the rhizosphere or the interior of the plant and promotes growth by increasing the supply or availability of primary nutrients to the host plant"<br><br>Vessey (2003)	"…any bacterial or fungal inoculants applied to plants with the aim to increase the availability of nutrients and their utilization by plants, regardless of the nutrient content of the inoculant itself"<br><br>du Jardin (2015)
Biofertilizer (cont'd…)	"products (carrier or liquid based) containing living or dormant microbes (bacteria, actinomycetes, fungi, algae) alone or in combination, which help in fixing atmospheric N or solubilizers soil nutrients in addition to the secretion of growth promoting substances for enhancing crop growth and yield"<br><br>Dineshkumar et al. (2018)	products composed of beneficial microorganisms, either in their active form or inactive forms, formulated with a single strain or in a mixed form, capable of colonizing the rhizosphere or the internal tissues of plants<br><br>(Fasusi et al. 2021; Ibáñez et al. 2023)	formulation of living or latent cells of microbes, which provides additional advantage in nutrient uptake and plant performance in rhizosphere and the authors divide them into six different groups: (i) N-fixing microbes, (ii/iii) phosphorus (P)/K solubilizing and mobilizing microbes, (iv) zinc solubilizing microbes, (v) sulfur oxidizing microbes and (vi) plant promoting rhizobacteria.<br><br>Kumar et al. (2021)
Biofertilizer (cont'd…)	organic products that contain specific microorganisms obtained from plant roots and root zones and they colonize the environment of the rizosphere and the interior	a wide range of products, containing living or dormant microorganisms and helps to improve the growth and yield of administered crops	

Supplementary information

	of the plant to promote plant growth.<br><br>Daniel et al. (2022)	Shahwar et al. (2023),	
Biostimulant	In reference to plants, a biostimulant is a material which contains substance(s) and/or microorganisms whose function, when applied to plants or the rhizosphere, is to stimulate natural processes to enhance/benefit nutrient uptake, nutrient efficiency, tolerance to abiotic stress, or crop quality<br><br>O'Callaghan et al. (2022)	products that include substances produced by microorganisms or other beings<br><br>Mitter et al. (2021).	"…any substances or microorganisms applied to plants with the aim to enhance nutrition efficiency, abiotic stress tolerance and/or crop quality traits, regardless of its nutrients content"<br><br>du Jardin (2015)
Biostimulant (cont'd…)	a substance, microorganism, or mixture thereof, that, when applied to seeds, plants, the rhizosphere, soil, or other growth media, act to support a plant's natural processes independently of the biostimulants nutrient content, thereby improving nutrient availability, uptake or use efficiency, tolerance to abiotic stress, and consequent growth, development, quality, or yield<br><br>BPIA (2023)		
Biopesticide	biological products to combat pests	microorganisms that promote plant growth through biofungicidal effects, bionematocides, bioinsecticides or any other products with similar activity that favor plant health	products enriched with microbial inoculants capable of overcoming the stress caused due to phytopathogens and can promote the health of the crop

Supplementary information

	Mitter et al. (2021)	Vessey (2003).	Yadav and Sarkar (2019).
Bioinoculant	bioinoculants - preparations containing single or consortia of microorganisms when introduced to the crop, and are dedicated to carrying out particular functions like, growth promotion or biocontrol<br><br>Maitra et al. (2021)		
Enhancer	substances that enhance plant growth, health, and productivity or provide other direct or indirect benefits to a plant's development.<br><br>Mercier (2023)		

The definitions in **Table S1** are visualized using a word cloud for term "biofertilizer" (**Fig S1A**) and "biostimulant (**Fig S1B**). As is showed in Figure S1 larger text indicates more frequeny use of a term and colors are used simply to establish a visual gradient. In biofertilizer (Fig S1A) is highlighted the words plant, microbes or microorganisms, growth, nutrients, and rhizospher and these words are fundamental to the definition and function of biofertilizers. Biostimulants in Figure S1B are more related to the words plants, nutrient, tolerance, quality, abiotic, applied, stress, microorganisms, showing the broader meaning of the word, which is why, in many cases, biofertilizer is included in the definition of biostimulants.

Supplementary information

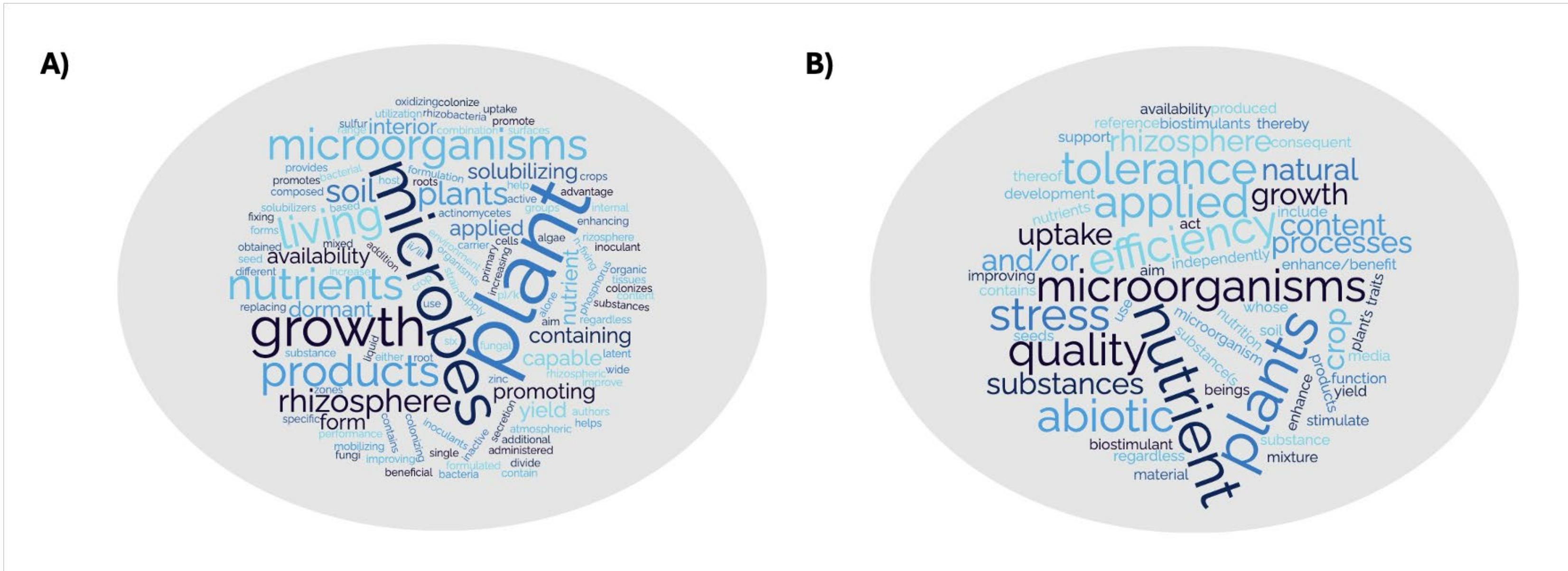

Figure S1. Word cloud visualization of definitions for the terms **A)** “biofertilizer” and **B)** “biostimulant.

2. Syntactic analysis of select peer reviewed articles

To gain a deeper understanding, the following manuscripts were identified as text which defines key terms in article.

1. Aloo et al. (2022);
2. Chaudhary et al. (2022);
3. Daniel et al. (2022);
4. Dineshkumar et al. (2018);
5. du Jardin (2015);

Supplementary information

6. Fasusi et al. (2021);
7. Ibáñez et al. (2023);
8. Kumar et al. (2021);
9. Malusá and Vassilev (2014);
10. Mitter et al. (2021);
11. Nosheen et al. (2021);
12. O'Callaghan et al. (2022);
13. Okon and Labandera-Gonzalez (1994);
14. Patel et al. (2023);
15. Shahwar et al. (2023);
16. Somers et al. (2004);
17. Vessey (2003).

Each document was downloaded, and then the a Python script was used to analyze the term (i.e., token) frequency using the RegExp model [1]. First, each PDF was cleaned and normalized (casing, splitting, whitespace correction, symbol filtration and stop word filtering). The list of RegExp tokens (excluding stop words, symbols and numbers) contained 195,054 tokens (**Fig S2**).

Supplementary information

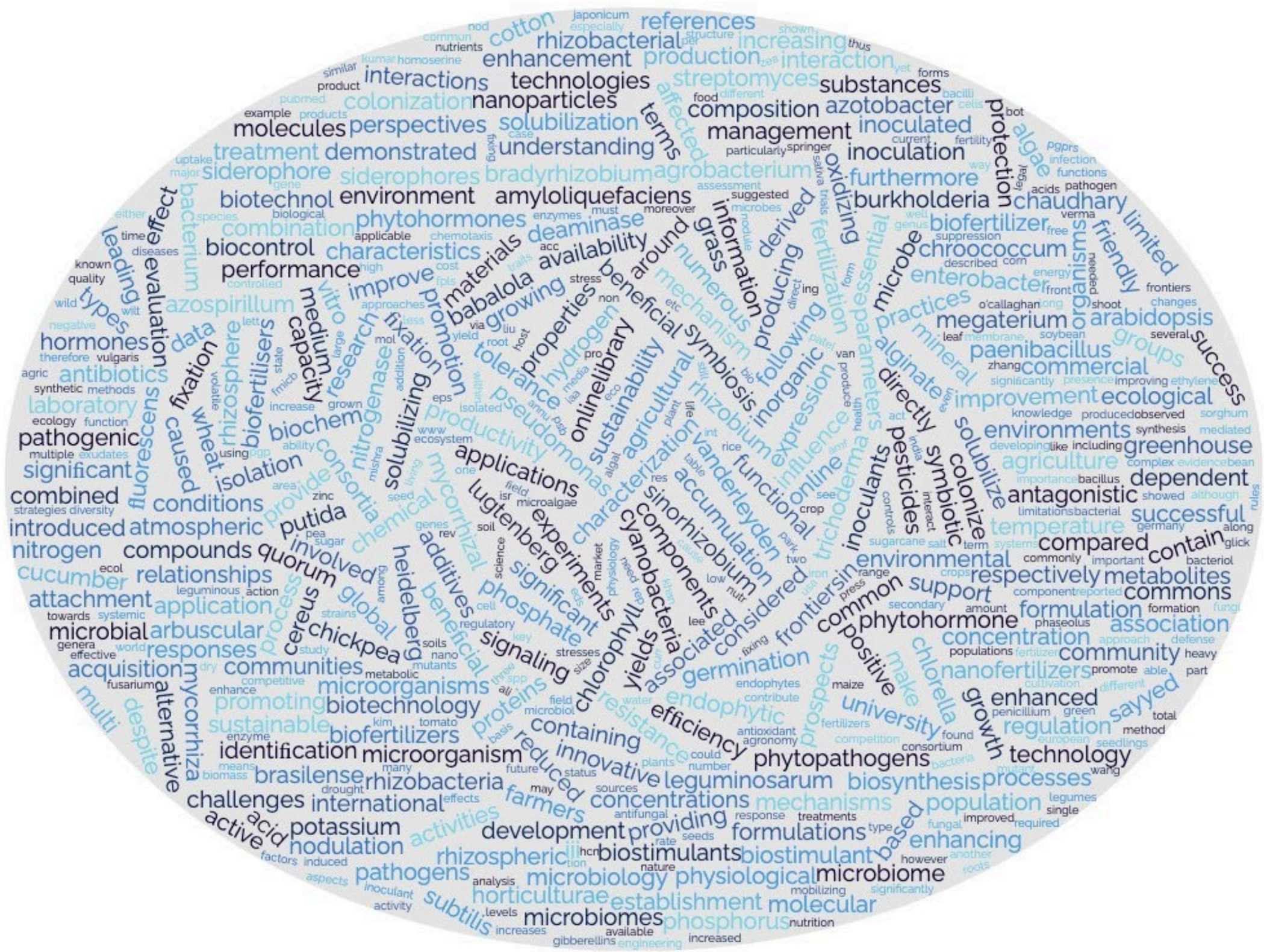

Figure S2. All tokens from PDFs (excluding stop words and symbols).

The data is also visualized using a frequency distributionin **Fig S3**. The term “biofertilizer” is highlighted in red/bold font. The inset shows the top 50 tokens in the frequency analysis.

Supplementary information

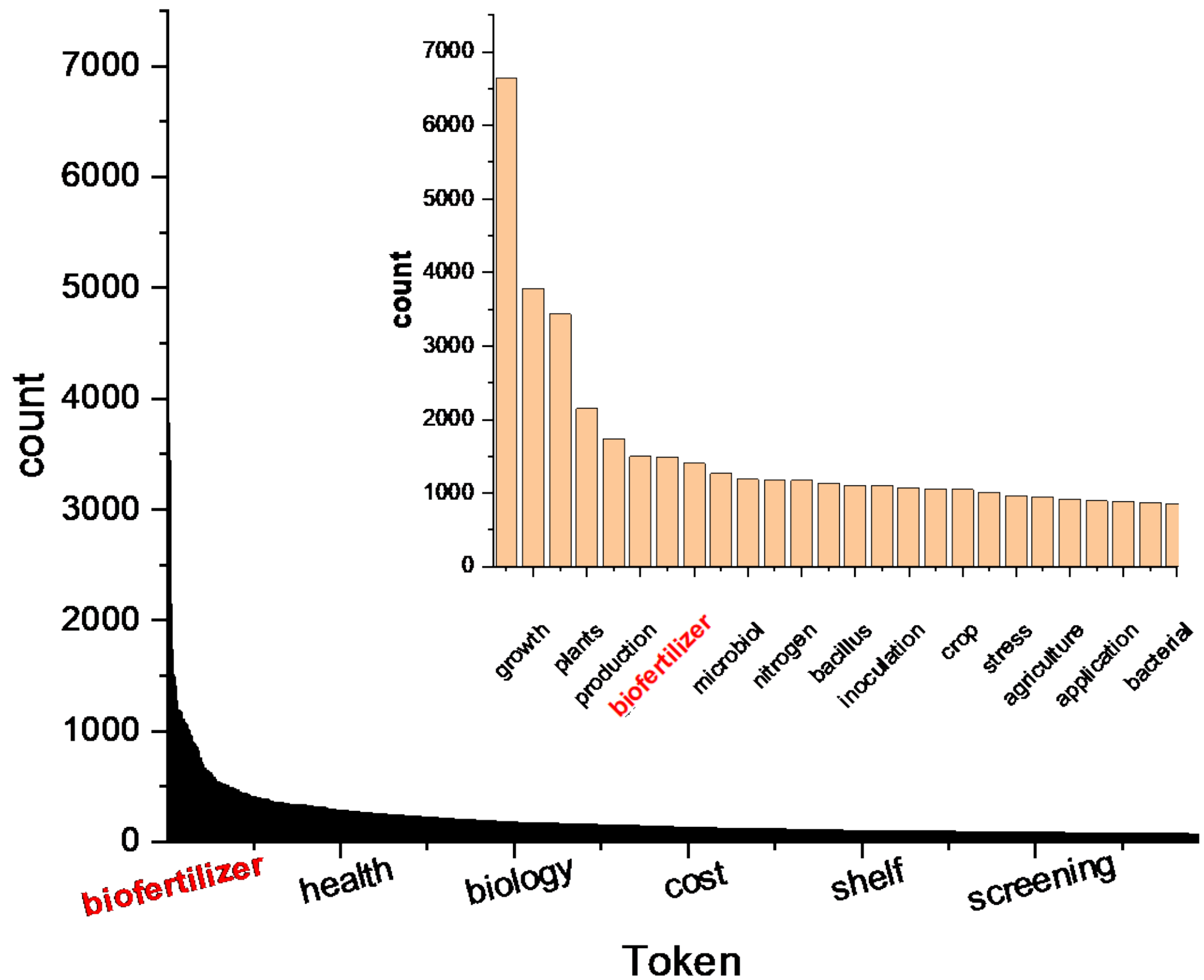

Figure S3. RegExp Token frequency analysis for select manuscripts related to biofertilizer definition. Inset shows top 50 tokens based on frequency count.

Supplementary information

Data were further analyzed by creating a merged list of RegExp tokens. As shown in **Table S2**, similar terms were merged. The token with highest use frequency is noted as "token", and syonymous terms are noted in concatentated columns. For brevity, only the top 30% of high frequency tokens were merged. The merged list of tokens consists of 58,740 tokens from the select PDF files.

Table S2. List of top 30% of tokens (thematically combined). High frequency tokens with no similar terms or synonyms are not shown in the table, but were included in the database for analysis.

**token**	**terms merged(1)**	**terms merged(2)**	**terms merged(3)**	**terms merged(4)**
plant	plants			
microbial	microbiol	microorganisms	microbes	strains
soil	soils			
growth	grown			
biofertilizers	biofertilizer	fertilizers		
root	roots			
promoting	promotion			
inoculation	inoculants			
bacteria	bacterial			
agriculture	agricultural	agric		
crop	crops			
production	products			
application	applied	applications		
yield	yields			
yield	yields			
solubilizing	solubilization	soluble		
nutrient	nutrients			
stress	stresses			
phosphate	phosphorus			
fungi	fungal	fungus		

sustainable	sustainability			
development	developing			

Figure S4 shows a frequency distribution plot for the merged tokens. The term “biofertilizer” is within the top five high frequency tokens, and has a similar use ocunt as: “plant”, “microbial”, “bacteria”, “soil”, “growth”, and “root” (Figure S5).

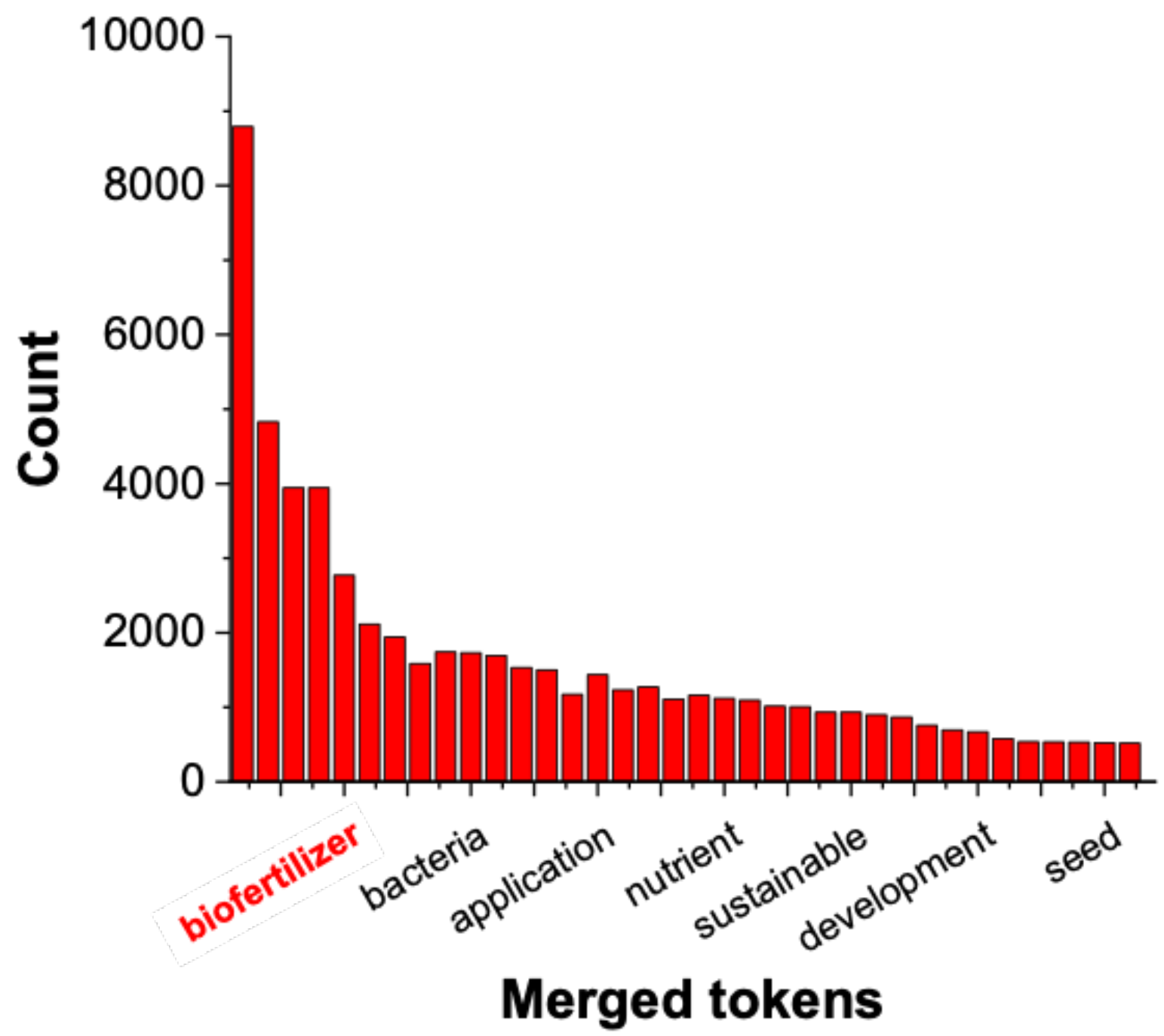

Figure S4. Mered RegExp Token frequency analysis for select manuscripts related to biofertilizer definition.

Supplementary information

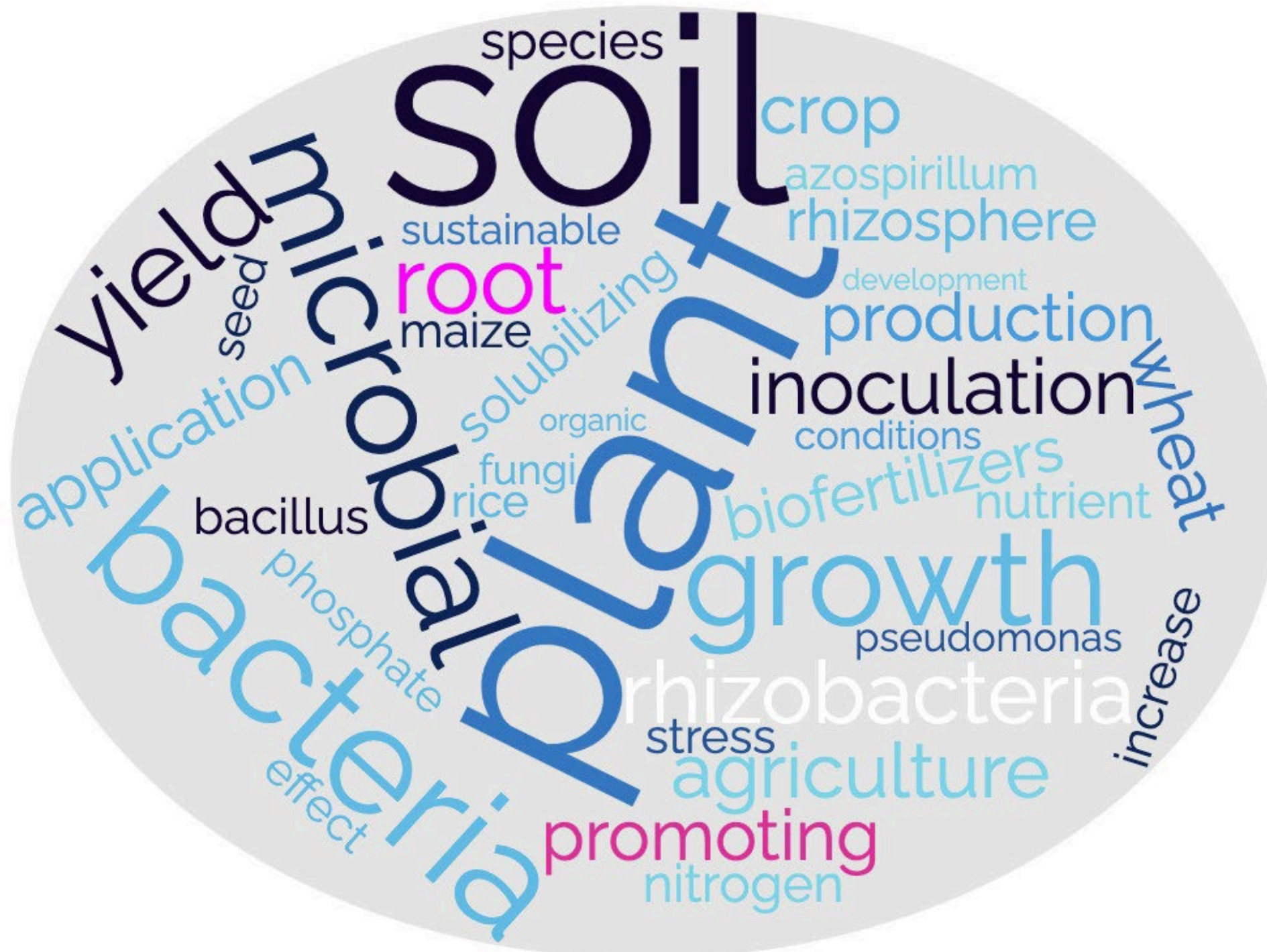

Figure S5. Merged token frequency based on histogram in Fig S4.

3. Possible synonyms and antonyms identified in peer reviewed literature

Supplementary information

According to the literature, many terms are synonymous (**Table S3**). A detailed study to identify synsets based on semantic analysis with largue language models is needed to confirm these relationship(s).

Table S3. Synonym and antonym sets (i.e., synsets) for key terms according to published literature.

**Term**	**Synonym** (Source)	**Synonym** (Source)	**Synonym** (Source)	**Antonym** (Source)	**Antonym** (Source)
Biofertilizer	(bio)inoculant<br>Fasusi et al. (2021); Ibáñez et al. (2023)	bioformulation<br>Fasusi et al. (2021); Ibáñez et al. (2023)	biostimulant<br>du Jardin (2015); Mercier (2023); (BPIA 2023)	organic fertilizer<br>Malusá and Vassilev (2014)	mineral fertilizer<br>Malusá and Vassilev (2014)
Biostimulant	microbial inoculant<br>O'Callaghan et al. (2022)	biofertilizer<br>du Jardin (2015); Mercier (2023); (BPIA 2023)	enhancer<br>Mercier (2023),		
Biocontrol	(bio)inoculant<br>Maitra et al. 2021; O'Callaghan et al. (2022)				
Biopesticide	biofungicidal<br>Vessey (2003)	bionematocides<br>Vessey (2003)	bioinsecticides<br>Vessey (2003)		

Supplementary information

4. Discussion/Limitations

- No semantic analysis performed (only syntax)
- Phosphate is low on the list (may be different if literature review focused more on AMF)
- Although beyond the scope of this work, the analysis showed that the term “biofertilizer” is semantically related to "enhancer", “stimulator”, and a few other terms. This show that is important to continue the work.
